# The Senokine Gas6 Promotes Aortic Valve Calcification Through CaMKII–DRP1–Mediated Terminal Scission of Mitochondrial‐Derived Vesicles

**DOI:** 10.1111/acel.70758

**Published:** 2026-10-20

**Authors:** Yaoyu Zhang, Jialiang Zhang, Yanjiani Xu, Wenhua Lei, Fei Xu, Junli Li, Zisong Wei, Bo Su, Shuang Liu, Jing Zhou, Hongde Li, Junpeng Ran, Weiya Li, Tuerhongjiang Tuerdi, Xiaojiao Wang, Zhi Fang, Fangyang Huang, Mao Chen

**Affiliations:** ^1^ Laboratory of Cardiac Structure and Function, Institute of Cardiovascular Diseases, West China Hospital Sichuan University Chengdu People's Republic of China; ^2^ Department of Cardiology, West China Hospital Sichuan University Chengdu People's Republic of China; ^3^ Cardiac Structure and Function Research Key Laboratory of Sichuan Province, West China Hospital Sichuan University Chengdu People's Republic of China; ^4^ Core Facilities of West China Hospital Chengdu Sichuan China; ^5^ Department of Cardiovascular Surgery, West China Hospital Sichuan University Chengdu People's Republic of China

**Keywords:** aortic valve calcification, calcific aortic valve disease, Gas6, mitochondrial‐derived vesicles

## Abstract

Calcific aortic valve disease (CAVD) is an age‐associated valvular disorder in which calcification expands from discrete foci into adjacent tissue. We investigated whether mitochondrial‐derived vesicles (MDVs) released by human valvular interstitial cells (hVICs) exert pro‐calcific effects on recipient cells and how MDV release is regulated. Using ex vivo hemodynamic perfusion of human CAVD tissue, multi‐omics profiling, live‐cell single‐organelle tracking, MDV transfer assays, and in vivo Gas6 deletion, we found that calcifying hVICs hypersecreted MDVs that exerted pro‐calcific effects in recipient hVICs. High‐resolution imaging showed that mitochondria remodeled into donut‐shaped structures under pro‐osteogenic conditions; these structures served as physical platforms for MDV budding. Human CAVD valve tissues and hVICs treated with pro‐osteogenic medium also exhibited senescence‐associated features. Multi‐omics profiling identified growth arrest‐specific 6 (Gas6), a senokine, among 36 concordantly upregulated candidates. Functional analyzes showed that Gas6 promoted terminal MDV scission while leaving donut‐shaped mitochondrial precursor formation intact. Mechanistically, Gas6 promoted intracellular Ca^2+^ accumulation, calcium/calmodulin‐dependent protein kinase II (CaMKII) activation, and dynamin‐related protein 1 (DRP1) phosphorylation at Ser616, supporting a Ca^2+^–CaMKII–DRP1 axis that facilitates terminal MDV scission. Pharmacological inhibition of CaMKII or DRP1 reduced MDV hypersecretion and subsequent recipient‐cell calcification, and Gas6 deletion in vivo attenuated valvular stenosis, calcification, and fibrotic remodeling. These findings identify Gas6‐dependent terminal MDV scission as a mechanism contributing to the intercellular propagation of valvular calcification in a CAVD context characterized by senescence‐associated features.

## Graphical abstract

In age‐associated calcific aortic valve disease (CAVD), human valve tissues and calcifying human valvular interstitial cells (hVICs) exhibit senescence‐associated features, while calcifying hVICs hypersecrete mitochondrial‐derived vesicles (MDVs). Donut‐shaped mitochondria serve as physical platforms for MDV budding, whereas Gas6 promotes Ca^2+^–CaMKII–DRP1‐dependent terminal scission without altering precursor formation. MDV preparations from calcifying hVICs exert pro‐calcific effects on recipient hVICs, supporting Gas6‐regulated MDV release as one mechanism contributing to valvular calcification.

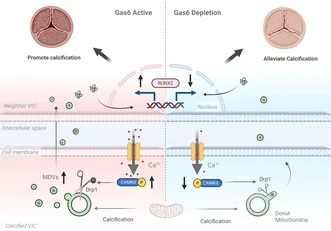

## 1 Introduction

Calcific aortic valve disease (CAVD) is an age‐associated valvular disorder and the most prevalent valvular heart disease among older adults, affecting more than 25% of individuals over 65 years of age (Coffey et al. 2021; Kraler et al. 2022). No pharmacological therapy has been shown to halt or reverse disease progression (Blaser et al. 2025; Praz et al. 2025). Human valvular interstitial cells (hVICs), the resident cell population of the aortic valve, are central to CAVD pathogenesis (Benkhoff et al. 2025; Kraler et al. 2022). Senescence‐associated changes and mitochondrial dysfunction have been observed in calcifying valve tissue and hVIC models and may shape calcification (Greenberg et al. 2022; Kroemer et al. 2025; Liu, Xu, et al. 2024; Qian et al. 2026).

A clinically significant feature of valvular calcification is its spatial progression: calcification typically begins at discrete foci and extends into adjacent tissue (Dweck et al. 2014; Otto et al. 1994; Qin and Bäck 2025). This point‐to‐surface pattern raises the possibility that calcifying cells influence neighboring hVICs through extracellular mediators rather than remaining passive endpoints. The identities of these mediators and the mechanisms that regulate their release remain unclear.

Mitochondrial‐derived vesicles (MDVs), membranous vesicles roughly 70–150 nm in diameter, have recently been recognized as a mitochondrial quality control mechanism and as intercellular messengers under cellular stress (Liu, Wang, et al. 2024; Picca et al. 2019; Sugiura et al. 2014). MDVs are generated through spatially targeted budding that selectively encapsulates damaged mitochondrial proteins, lipids, and mitochondrial DNA (mtDNA) (Iorio et al. 2024; Lou et al. 2025). They are heterogeneous and can be grouped into subpopulations based on cargo composition, which reflects the nature and severity of the inciting cellular stress (König and McBride 2024; Soubannier et al. 2012). Single‐positive MDVs carrying exclusively outer mitochondrial membrane markers (TOM20^+^/PDH^−^) or exclusively matrix components (TOM20^−^/PDH^+^) are constitutively generated under basal conditions or mild oxidative stress (Todkar et al. 2021). Double‐positive MDVs harboring both outer membrane and matrix constituents (TOM20^+^/PDH^+^) arise under more severe metabolic dysfunction or strong oxidative stress, and have been linked to disease pathogenesis (Sugiura et al. 2014; Tang et al. 2025). MDVs can be secreted into the extracellular space under pathological conditions (Phinney et al. 2015). Extracellular MDVs have been reported to influence recipient‐cell function (Iorio et al. 2024).

Whether hVIC‐derived extracellular MDVs contribute to CAVD by exerting pro‐calcific effects on neighboring cells, and how their final separation from donor mitochondria is controlled, remain unknown.

In this study, we combined ex vivo perfusion of human aortic valves, primary hVIC models, multi‐omics profiling, live‐cell single‐organelle tracking, MDV transfer assays, and in vivo validation to investigate the release and pro‐calcific activity of MDVs in CAVD. We found that CAVD valve tissue and hVICs treated with pro‐osteogenic medium released increased levels of MDVs, and that pathological MDV preparations increased mineral deposition and osteogenic marker expression in recipient hVICs. Multi‐omics analysis identified growth arrest‐specific 6 (Gas6), recently described as a candidate senoprotein (Ding et al. 2025), for mechanistic investigation. Live‐cell imaging and pathway perturbation showed that Gas6 promotes Ca^2+^–CaMKII–DRP1‐dependent terminal scission of MDVs from donut‐shaped mitochondrial precursors without being required for precursor formation. Systemic Gas6 deletion attenuated injury‐induced aortic valve stenosis and calcification in vivo. Together, these findings identify Gas6‐dependent terminal MDV scission as a mechanism that may contribute to the intercellular expansion of valvular calcification.

## 2 Methods

Detailed experimental procedures are provided in the Supporting Information S1.

### 2.1 Human Samples and Ethics

Human aortic valve tissues were obtained from 24 patients aged 60–73 years, including 12 patients undergoing surgical aortic valve replacement for severe CAVD and 12 age‐ and sex‐matched patients undergoing cardiac surgery for pure aortic regurgitation without valve calcification (nonCAVD controls). The mean ages of the nonCAVD and CAVD groups were 64.42 ± 2.61 and 65.42 ± 3.96 years, respectively (mean ± SD; *p* = 0.474). Six patients per group provided valve tissue for ex vivo hemodynamic perfusion and tissue‐derived MDV experiments. The remaining six patients per group provided tissue for human histological and molecular analyzes, including assessments of senescence‐associated features. Primary hVICs used in all in vitro experiments were isolated exclusively from the six nonCAVD donors in the second patient subset. Patients with chronic kidney disease (estimated glomerular filtration rate < 60 mL/min/1.73 m^2^) were excluded. Calcified and non‐calcified regions within each CAVD valve leaflet were identified based on preoperative computed tomography (CT) and distinguished primarily by gross visual inspection, with stereomicroscopic examination used when necessary. Regions containing calcific nodules were classified as calcified, whereas regions with normal leaflet thickness and no apparent calcification were classified as non‐calcified. This study was approved by the Ethics Committee of West China Hospital (Approval No. 2022–1874). The study was conducted in accordance with the Declaration of Helsinki, and written informed consent was obtained from all participants before tissue collection.

### 2.2 Sex Considerations

Both male and female patients were included in the human tissue cohort. All in vitro experiments were independently replicated using primary hVICs derived from six separate patient donors of both sexes. For in vivo studies, the aortic wire injury model included both male and female mice. The diet‐induced ApoE^−/−^ model used male mice only.

### 2.3 Primary hVIC Culture and Pro‐Osteogenic Medium Treatment

Primary hVICs were isolated from non‐calcified aortic valves by collagenase digestion and maintained in Dulbecco's modified Eagle's medium (DMEM) with 10% fetal bovine serum. Cells between passages 3 and 6 were used for all experiments unless otherwise indicated; the passage‐comparison experiment used hVICs at P1, P3, and P7. For treatment with pro‐osteogenic medium (OM), hVICs were cultured in OM containing 10 mM β‐glycerophosphate, 50 μg/mL ascorbic acid, and 10 nM dexamethasone; control cells were maintained in control medium (CM). Except for the time‐course experiment, in which hVICs were analyzed after 3, 7, and 14 days, cells in all other CM and OM experiments were cultured for 21 days.

### 2.4 Extracellular Vesicle and MDV Isolation

Extracellular vesicles (EVs) were isolated from conditioned media or ex vivo perfusate by differential ultracentrifugation and characterized by nanoparticle tracking analysis and immunoblotting for EV markers. The MDV subpopulation was quantified by nano‐flow cytometry using fluorescein isothiocyanate (FITC)‐conjugated anti‐TOM20 antibody. For functional studies, MDVs were enriched by immunoprecipitation with TOM20‐conjugated magnetic beads.

### 2.5 Ex Vivo Hemodynamic Simulation

Freshly harvested valve leaflets of equal wet weight were mounted in a custom pulsatile flow chamber perfused at 1 Hz to simulate physiological cardiac cycling. After 6 h of perfusion, the circulating medium was collected for vesicle isolation.

### 2.6 MDV Functional Transfer Assay

Purified MDVs from donor hVICs or valve tissues were applied to naïve recipient hVICs maintained in control medium. Recipient cells were cultured for 21 days, after which calcification and osteogenic marker expression were assessed.

### 2.7 Multi‐Omics Profiling

Isobaric tags for relative and absolute quantitation (iTRAQ)‐based proteomic and bulk RNA transcriptomic data from human CAVD and nonCAVD valve tissues were obtained from our previously published dataset (Shao et al. 2026). Differentially expressed candidates were identified by integrating the proteomic and transcriptomic datasets.

### 2.8 Gene Silencing, Overexpression, and RNA Sequencing

Gas6 was silenced using siRNA (siGas6) or overexpressed via adenoviral transduction (AdGas6). RNA sequencing was performed on siCtrl and siGas6 hVICs under osteogenic conditions, followed by gene set enrichment analysis (GSEA) and Gene Ontology (GO) enrichment analyzes. Pharmacological inhibition of DRP1 (Mdivi‐1, 10 μM) and CaMKII (KN‐93, 10 μM) was employed where indicated.

### 2.9 Live‐Cell Imaging

Mitochondrial dynamics were visualized in hVICs stained with PKmito Deep Red using time‐lapse confocal microscopy at 25‐s intervals. Time‐resolved three‐dimensional reconstructions were generated from sequential confocal z‐stacks acquired at each time point using Imaris software (version 10.1.1). Donut‐shaped mitochondria and MDV budding events were quantified manually by blinded observers.

### 2.10 Animal Models

Wild‐type C57BL/6J and systemic Gas6‐knockout (Gas6^−/−^) mice (8–10 weeks old, both sexes, *n* = 6 per group) underwent aortic wire injury or sham surgery, and disease progression was evaluated at 4 weeks by echocardiography and histological analysis. In a separate dietary model, ApoE^−/−^ mice (8 weeks old, male, *n* = 6 per group) were fed a high‐fat diet supplemented with 1.25% cholesterol and 0.5% sodium cholate for 24 weeks. All surgical procedures were performed under isoflurane anesthesia (3% induction, 1.5%–2% maintenance in room air). Anesthetic depth was monitored throughout by pedal withdrawal reflex. Post‐operative analgesia was provided by subcutaneous meloxicam (5 mg/kg). At experimental endpoints, deep anesthesia was confirmed by loss of pedal reflex, followed by cervical dislocation as the method of euthanasia in accordance with the Guidelines for the Euthanasia of Animals: 2020 Edition issued by the American Veterinary Medical Association (2020). All animal procedures were approved by the Institutional Animal Care and Use Committee of West China Hospital (Approval No. 20240311055).

### 2.11 Statistical Analysis

Normality was verified by the Shapiro–Wilk test. Two‐group comparisons were performed using two‐tailed unpaired Student's *t*‐test. Categorical variables were compared using Fisher's exact test. Multi‐group comparisons were performed by one‐way ANOVA with Tukey's post hoc test. Sex‐by‐treatment interactions were assessed using two‐way ANOVA. Detailed definitions of biological replicates, sex‐stratified analyzes, and sample sizes for each experiment are provided in the Supporting Information S1.

## 3 Results

### 3.1 Mitochondrial‐Derived Vesicles Act as Pro‐Calcific Mediators in Valvular Calcification

To investigate whether MDVs contribute to CAVD pathogenesis, we developed an ex vivo hemodynamic simulation system to assess the release of vesicles from human aortic valve tissue under physiological flow conditions (Figure 1A). Aortic valve leaflets were obtained from patients undergoing surgical valve replacement for severe CAVD (*n* = 6). Age‐ and sex‐matched patients with non‐calcified aortic regurgitation served as controls (nonCAVD, *n* = 6) (Table S1).

**FIGURE 1:**
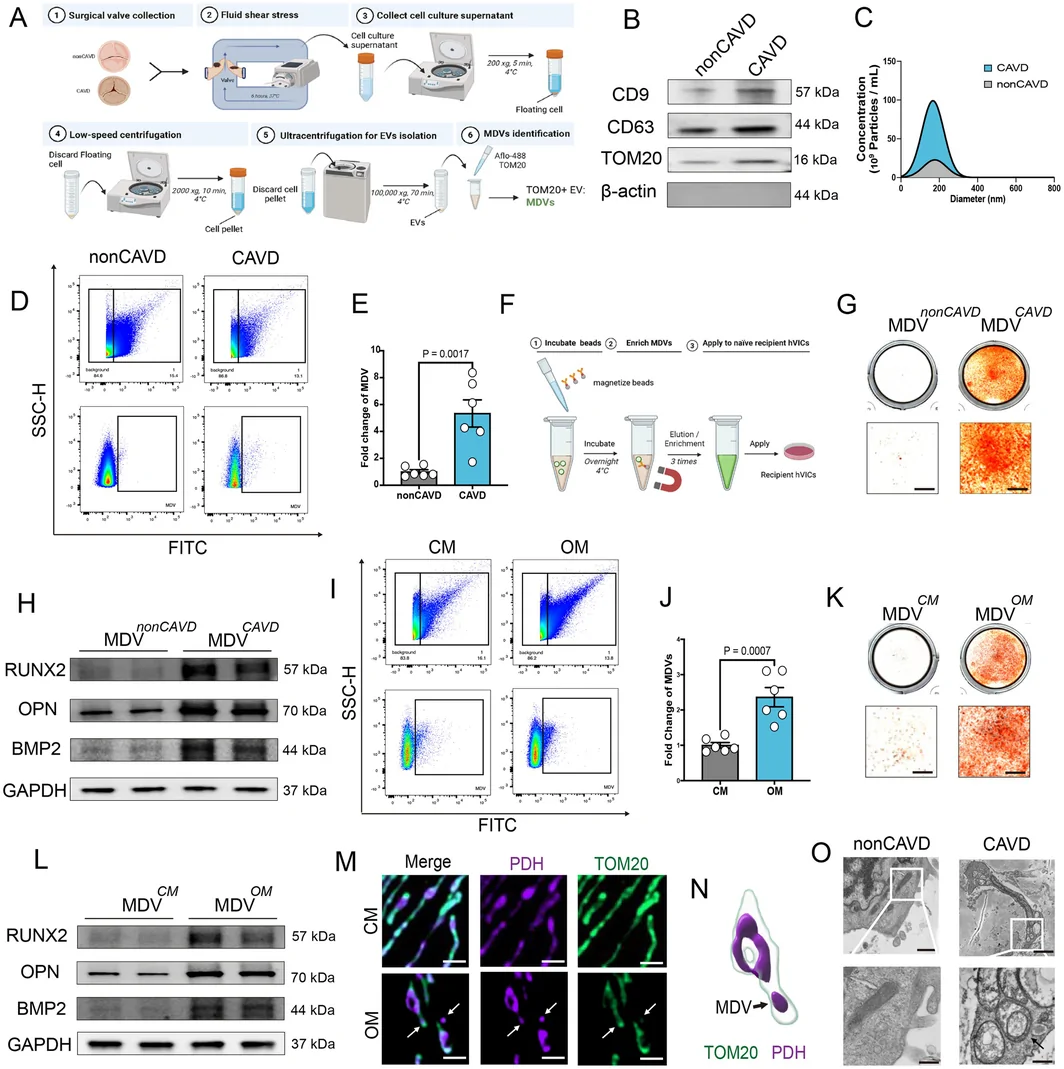
Mitochondrial‐derived vesicles act as pro‐calcific mediators in valvular calcification. (A) Schematic illustration of the ex vivo hemodynamic simulation system for vesicle collection from human aortic valve leaflets under physiological pulsatile flow conditions. (B) Immunoblot analysis of EV markers (CD9, CD63), mitochondrial outer membrane protein TOM20, and cytoplasmic marker β‐actin (negative control) in EVs isolated from the perfusate of non‐CAVD and CAVD valve leaflets. (C) Nanoparticle tracking analysis (NTA) showing the size distribution of isolated EVs from nonCAVD and CAVD perfusates. (D and E) Representative nano‐flow cytometry plots (D) and quantification (E) of TOM20‐positive mitochondrial‐derived vesicles (MDVs) in EVs isolated from nonCAVD and CAVD valve perfusates. (F) Schematic illustration of the immunoadsorption‐based MDV enrichment strategy using TOM20‐conjugated magnetic beads. (G) Alizarin Red S staining (scale bar, 200 μm) of naïve recipient hVICs following treatment with MDV preparations enriched from nonCAVD (MDV^nonCAVD^) or CAVD (MDV^CAVD^) valve tissues. (H) Immunoblot analysis of RUNX2 (runt‐related transcription factor 2), OPN (osteopontin), and BMP2 (bone morphogenetic protein 2) in recipient hVICs treated with MDV^nonCAVD^ or MDV^CAVD^. GAPDH served as the loading control. See Figure S1A for densitometric quantification. (I and J) Representative nano‐flow cytometry plots (I) and quantification (J) of TOM20‐positive MDVs in conditioned media from hVICs cultured under control medium (CM) or pro‐osteogenic medium (OM) conditions. (K) Alizarin Red S staining (scale bar, 200 μm) of naïve recipient hVICs following treatment with MDV preparations derived from CM‐ or OM‐cultured hVICs. (L) Immunoblot analysis of RUNX2, OPN, and BMP2 in recipient hVICs treated with MDV^*CM*^ or MDV^*OM*^. GAPDH served as the loading control. See Figure S1E for densitometric quantification. (M) Representative immunofluorescence co‐staining images of TOM20 (green) and PDH (pyruvate dehydrogenase, magenta) in hVICs cultured under CM or OM conditions. Arrows indicate TOM20^+^/PDH^+^ vesicular buds contiguous with donut‐shaped mitochondrial structures. Scale bars, 1.2 μm. (N) Three‐dimensional reconstruction of confocal z‐stacks from an OM‐treated hVIC stained for TOM20 (green) and PDH (magenta), showing a closed, donut‐shaped mitochondrial structure with an attached TOM20^+^/PDH^+^ vesicular bud. A rotational view of the reconstructed volume is provided in Video S1. (O) Representative TEM images of aortic valve leaflets from nonCAVD and CAVD patients. Lower panels show higher‐magnification views of the boxed regions; a donut‐shaped mitochondrial profile is evident in the lower CAVD panel, whereas no comparable profile is evident in the lower nonCAVD panel. Scale bars, 1 μm (upper panels) and 0.5 μm (lower panels). A multi‐angle rotational view of the corresponding three‐dimensional TEM reconstruction is provided in Video S2. Data are presented as mean ± SEM (*n* = 6 independent experiments or patients per group). For densitometric quantification, protein expression levels were normalized to the first group. *p* values were determined by two‐tailed unpaired Student's *t*‐test.

Following 6 h of pulsatile perfusion of equal wet weights of valve tissue from each patient, the circulating perfusate was collected and subjected to differential ultracentrifugation to isolate extracellular vesicles (EVs). Immunoblot analysis confirmed the expression of classical EV markers (CD9 and CD63) alongside the mitochondrial outer membrane protein TOM20, while the cytoplasmic marker β‐actin was undetected (Horbay et al. 2025) (Figure 1B), indicating successful EV isolation with effective depletion of cytoplasmic contaminants. Nanoparticle tracking analysis (NTA) showed that the isolated vesicles exhibited peak diameters of approximately 180 nm (Figure 1C), consistent with the expected size range of EVs (Horbay et al. 2025). To specifically quantify the MDV subpopulation within this total EV pool, the vesicles were labeled with a TOM20‐FITC antibody and analyzed by nano‐flow cytometry. Quantitative analysis revealed that CAVD valve leaflets released significantly higher levels of MDVs compared with non‐CAVD controls (Figure 1D,E).

We next examined whether MDVs released from calcified valves exert pro‐calcific effects on recipient cells. To enrich MDVs, we used an immunoadsorption‐based separation strategy with TOM20‐conjugated magnetic beads (Zhang et al. 2025) (Figure 1F). MDV preparations from CAVD or nonCAVD valve tissues were applied to naïve primary hVICs isolated from non‐calcified aortic valves and maintained in control medium. Alizarin Red S staining revealed that CAVD‐derived MDV preparations (MDV^*CAVD*^) induced substantial calcium deposition, whereas those from nonCAVD valves (MDV^*nonCAVD*^) produced minimal mineralization (Figure 1G). Immunoblot analysis showed that MDV^*CAVD*^ treatment increased the protein levels of RUNX2 (runt‐related transcription factor 2), OPN (osteopontin), and BMP2 (bone morphogenetic protein 2) compared with MDV^*nonCAVD*^ (Figure 1H; Figure S1A) (Rajamannan et al. 2011). These results show that CAVD‐derived MDV preparations promote mineral deposition and osteogenic marker expression in recipient hVICs.

Since the ex vivo perfusate was derived from intact valve tissue containing both interstitial and endothelial cells, and hVICs are the principal cell type undergoing osteogenic activation during valvular calcification (Rutkovskiy et al. 2017), we next established a pure hVIC culture system to determine whether hVICs themselves release MDVs under OM conditions. hVICs were cultured in either control medium (CM) or pro‐osteogenic medium (OM). Conditioned media were collected from equal numbers of cells in equivalent volumes and analyzed by nano‐flow cytometry. OM‐cultured hVICs released higher levels of MDVs compared with CM‐cultured cells (Figure 1I,J), recapitulating the elevated MDV release observed in human CAVD tissue. Increasing OM strength from 0.5× to 2.0× produced a dose‐dependent increase in MDV release (Figure S1C,D), showing that stronger OM treatment was accompanied by greater MDV release.

To assess the functional capacity of these in vitro‐generated MDVs, we performed an analogous MDV transfer assay (Figure S1B). MDV preparations isolated from equivalent volumes of conditioned medium were applied to naïve recipient hVICs maintained in control medium. Alizarin Red S staining confirmed that MDVs from OM‐cultured cells (MDV^*OM*^) induced extensive calcium deposition (Figure 1K). MDV^*OM*^ treatment also increased the protein levels of RUNX2, OPN, and BMP2 in recipient hVICs compared with MDVs from CM‐cultured cells (MDV^*CM*^) (Figure 1L; Figure S1E). These findings demonstrate that MDV preparations released by OM‐treated hVICs exert pro‐calcific effects on recipient cells.

To characterize the subtype of hVIC‐derived MDVs, we performed immunofluorescence co‐staining for TOM20 (mitochondrial outer membrane) and PDH (mitochondrial matrix) in hVICs (Tang et al. 2025). OM‐cultured cells exhibited TOM20^+^/PDH^+^ vesicular buds contiguous with mitochondrial structures, whereas such buds were not evident under CM conditions (Figure 1M), consistent with hVIC‐derived MDVs containing both mitochondrial outer‐membrane and matrix components—a subtype previously observed under conditions of severe mitochondrial dysfunction (Todkar et al. 2021). During immunofluorescence imaging, these MDV‐like buds were spatially associated with hollow, donut‐shaped mitochondria under OM conditions. Similar donut‐shaped mitochondria have been reported in primary murine osteoblasts, where they were implicated in MDV secretion (Suh et al. 2023). Three‐dimensional reconstruction of confocal z‐stacks from OM‐treated hVICs visualized a closed, donut‐shaped mitochondrial structure with an attached TOM20^+^/PDH^+^ vesicular bud (Figure 1N). A rotational view of the reconstructed volume is provided in Video S1.

This morphological remodeling was corroborated across multiple high‐resolution imaging modalities, including stimulated emission depletion (STED) super‐resolution microscopy (Figure S1F), transmission electron microscopy (TEM) (Figure S1G), and live‐cell imaging (Figure S1H). Quantitative analysis confirmed enrichment of donut‐shaped mitochondria in OM‐treated cells compared with controls (Figure S1I). In the representative TEM images shown, donut‐shaped mitochondrial profiles were evident in CAVD valve tissue but not in the nonCAVD control tissue (Figure 1O). Three‐dimensional TEM reconstruction of a representative CAVD profile supported its toroidal topology across multiple viewing angles (Video S2). Together, the three‐dimensional reconstructions provide spatial evidence for the toroidal morphology and its association with a vesicular bud, whereas live‐cell imaging provides complementary temporal evidence of MDV budding.

### 3.2 Senescence‐Associated Features and Gas6 Upregulation in CAVD

Having established increased MDV release and the pro‐calcific effects of MDV preparations derived from CAVD valve tissues and OM‐treated hVICs, we next characterized the source tissues and cells. Histological examination confirmed characteristic pathological changes in CAVD valves, including calcium deposition, as shown by von Kossa and Alizarin Red S staining, and collagen accumulation with fibrotic remodeling, as shown by modified Masson's trichrome and Picrosirius Red staining (Figure 2A).

**FIGURE 2:**
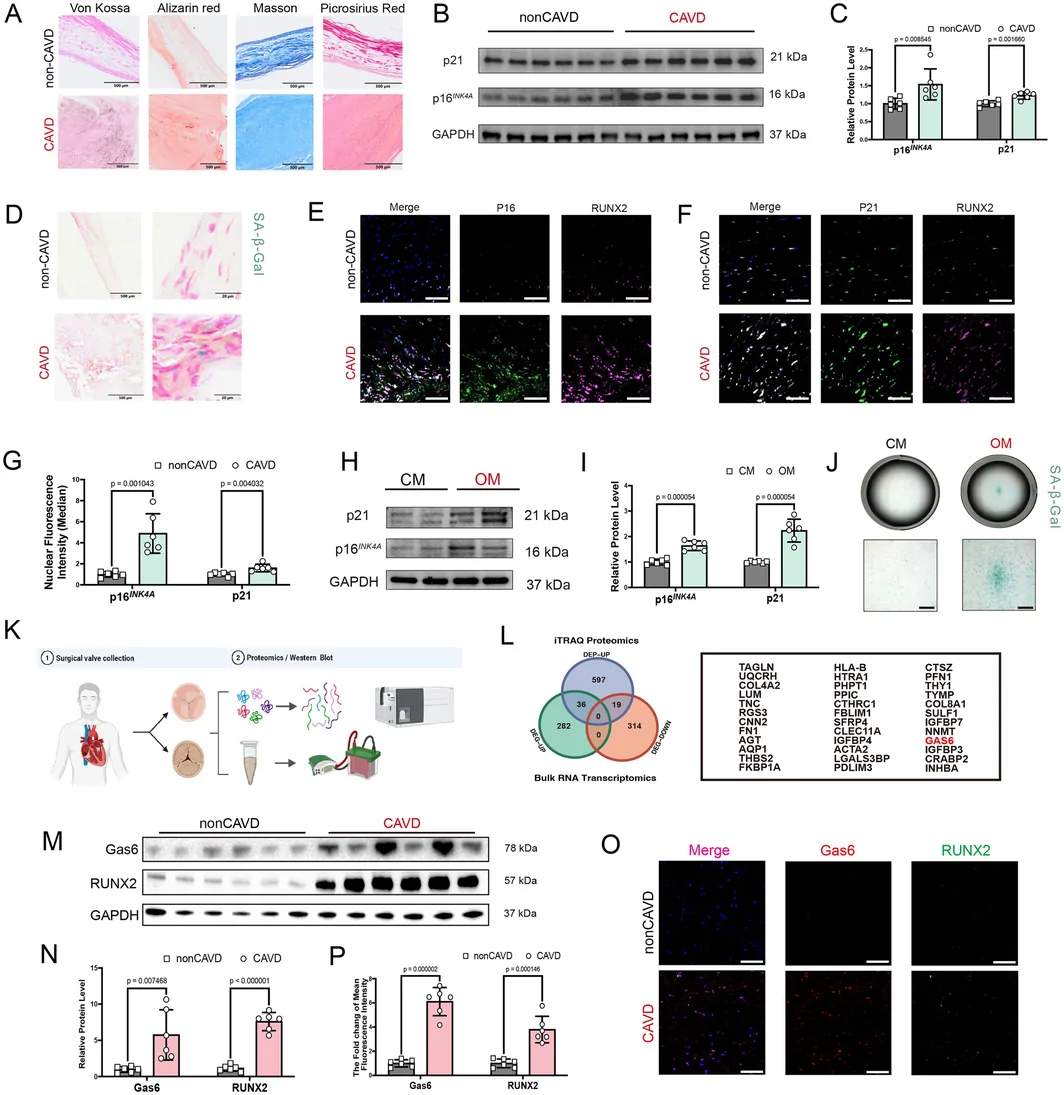
Senescence‐associated features and Gas6 upregulation in CAVD. (A) Representative histological images of nonCAVD and CAVD valve sections stained with von Kossa and Alizarin Red S to detect calcium deposition, and modified Masson's trichrome and Picrosirius Red to assess collagen deposition and fibrotic remodeling. Scale bars, 500 μm. (B, C) Representative immunoblots (B) and densitometric quantification (C) of p16^INK4A^ and p21 protein expression in nonCAVD and CAVD human valve tissues. GAPDH served as the loading control. (D) Representative low‐ and high‐magnification senescence‐associated β‐galactosidase (SA‐β‐gal) staining images of nonCAVD and CAVD human valve tissues. Scale bars, 500 μm (left) and 20 μm (right). (E–G) Representative immunofluorescence co‐staining images of p16^INK4A^ (green) and RUNX2 (magenta) (E), and p21 (green) and RUNX2 (magenta) (F), together with quantification of median nuclear p16^INK4A^ and p21 fluorescence intensity (G), in human non‐CAVD and CAVD valve leaflets. Nuclei were counterstained with DAPI (blue). Scale bars, 50 μm. (H and I) Representative immunoblots (H) and densitometric quantification (I) of p16^INK4A^ and p21 protein expression in hVICs cultured in control medium (CM) or pro‐osteogenic medium (OM). GAPDH served as the loading control. (J) Representative SA‐β‐gal staining images of CM‐ and OM‐treated hVICs. Lower panels show higher‐magnification views. Scale bar, 200 μm (lower panels). (K) Schematic overview of the multi‐omics screening strategy and subsequent molecular validation using human nonCAVD and CAVD valve tissues. (L) Venn diagram integrating iTRAQ (isobaric tags for relative and absolute quantitation)‐based proteomics and bulk RNA transcriptomics, illustrating the intersection of differentially upregulated proteins (DEP‐UP) and genes (DEG‐UP) and identifying 36 consistently upregulated candidates, including Gas6. (M, N) Representative immunoblots (M) and densitometric quantification (N) of Gas6 and RUNX2 protein expression in nonCAVD and CAVD valve tissues. GAPDH served as the loading control. (O and P) Representative immunofluorescence co‐staining images (O) and quantitative analysis of mean fluorescence intensity (P) for Gas6 (red) and RUNX2 (green) in human nonCAVD and CAVD valve leaflets. Nuclei were counterstained with DAPI (blue). Scale bar, 50 μm. Data are presented as mean ± SEM (*n* = 6 per group for human tissue analyzes; *n* = 6 independent experiments for hVIC analyzes). For densitometric quantification, protein expression levels were normalized to the first group. p16^INK4A^ and p21 immunofluorescence data are expressed as median nuclear fluorescence intensity; Gas6 and RUNX2 immunofluorescence data are expressed as mean fluorescence intensity and normalized to the first group. *p* values for all quantified between‐group comparisons were determined by two‐tailed unpaired Student's *t*‐test.

Given the age‐associated nature of CAVD, we next assessed whether the human valve tissues and OM‐treated hVICs used in this study exhibited senescence‐associated features. In human valve tissues, immunoblotting showed significantly higher p16^INK4A^ and p21 protein levels in CAVD than in nonCAVD samples (Figure 2B,C). Representative senescence‐associated β‐galactosidase (SA‐β‐gal) staining revealed positive foci within morphologically calcified regions of CAVD valves (Figure 2D). Quantification of dual immunofluorescence staining for p16^INK4A^/RUNX2 and p21/RUNX2 further showed significantly higher median nuclear fluorescence intensities of p16^INK4A^ and p21 in CAVD valve tissues (Figure 2E–G). Together, these findings support the presence of senescence‐associated features in human CAVD tissues.

To determine whether a similar context was present in the in vitro calcification model, we compared CM‐ and OM‐treated hVICs. OM‐treated hVICs showed significantly higher p16^INK4A^ and p21 protein levels than CM‐treated cells (Figure 2H,I), while representative SA‐β‐gal images showed qualitatively more pronounced staining under OM conditions (Figure 2J). Thus, OM treatment was accompanied by senescence‐associated features in hVICs. Similar marker changes were observed in recipient hVICs exposed to MDV preparations derived from OM‐treated donor hVICs or CAVD valve tissues, with higher p16^INK4A^ and p21 protein levels and qualitatively more pronounced SA‐β‐gal staining than in recipients exposed to the corresponding CM‐ or nonCAVD‐derived MDV preparations (Figure S2A–F).

With these tissue and cell‐model contexts established, we next used a separate disease‐comparison analysis to identify molecular candidates for functional testing in relation to the MDV phenotype (Figure 2K). Integration of our previously reported iTRAQ proteomic and bulk RNA transcriptomic datasets from human CAVD and nonCAVD valve tissues identified 36 candidates that were upregulated at both the transcript and protein levels, including GAS6 (Figure 2L; Shao et al. 2026).

GAS6 was recently identified as a candidate senoprotein in a human lifespan proteomic atlas (Ding et al. 2025). Within the age‐associated CAVD context established above, this prior annotation, together with GAS6's inclusion in the multi‐omics candidate set, provided additional biological rationale for prioritizing GAS6 for subsequent validation and functional testing. Immunoblotting independently confirmed higher Gas6 protein expression in CAVD tissues, paralleling RUNX2 upregulation (Figure 2M,N). Immunofluorescence staining showed greater Gas6 and RUNX2 signals in CAVD than in nonCAVD valve leaflets, with spatial overlap between the two proteins in CAVD tissue (Figure 2O,P). Similar increases in Gas6 and RUNX2 fluorescence were observed in aortic valve leaflets from ApoE^−/−^ mice fed a high‐fat diet compared with normal chow (Figure S2).

Together, the multi‐omics candidate identification and independent immunoblotting and immunofluorescence validation support higher Gas6 expression in CAVD.

### 3.3 Gas6 Promotes hVIC Calcification and Osteogenic Programming

We next tested whether Gas6 regulates hVIC calcification and osteogenic programming. We first examined Gas6 expression dynamics during pro‐osteogenic medium (OM) treatment in vitro using primary hVICs isolated from non‐calcified aortic valves (Figure S3A). Immunoblot analysis showed a progressive increase in Gas6 protein during OM treatment. Gas6 elevation was detectable by Day 3 and persisted through Day 14, paralleling increases in the osteogenic markers RUNX2, OPN, and BMP2 (Figure 3A–E). This temporal association prompted functional loss‐ and gain‐of‐function analyzes.

**FIGURE 3:**
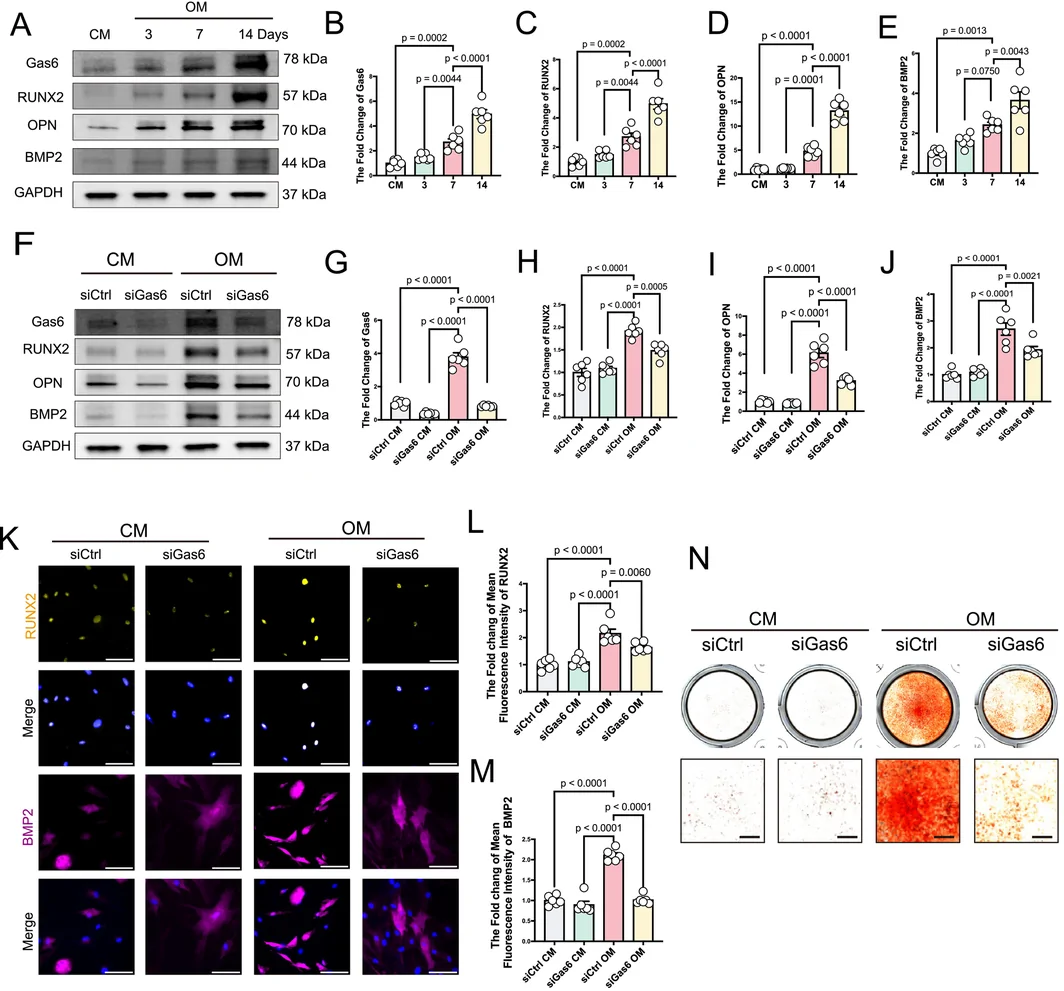
Gas6 promotes hVIC calcification and osteogenic programming. (A–E) Time‐course analysis of Gas6 and osteogenic marker expression during OM treatment. (A) Representative immunoblots of Gas6, RUNX2, BMP2, and OPN in hVICs cultured in control medium (CM) or pro‐osteogenic medium (OM) for 3, 7, and 14 days. GAPDH served as the loading control. (B–E) Densitometric quantification of Gas6 (B), RUNX2 (C), OPN (D), and BMP2 (E). (F–J) Gas6 and osteogenic marker expression in hVICs treated with siCtrl (non‐targeting control siRNA) or siGas6 (Gas6‐targeting siRNA) under CM and OM conditions. (F) Representative immunoblots of Gas6, RUNX2, OPN, and BMP2. GAPDH served as the loading control. (G–J) Densitometric quantification of Gas6 (G), RUNX2 (H), OPN (I), and BMP2 (J). (K–M) Immunofluorescence images and quantification of RUNX2 and BMP2 in siCtrl and siGas6 hVICs under CM and OM conditions. (K) Representative images of RUNX2 (yellow) and BMP2 (magenta) staining in siCtrl and siGas6 hVICs under CM and OM conditions. Nuclei were counterstained with DAPI (blue). Scale bars, 100 μm. (L, M) Quantification of mean fluorescence intensity of RUNX2 (L) and BMP2 (M). (N) Representative Alizarin Red S staining of siCtrl‐ and siGas6‐treated hVICs cultured in CM or OM for 21 days. Scale bar, 200 μm. Quantitative data are presented as mean ± SEM (*n* = 6 independent experiments). For densitometric and mean fluorescence intensity quantification, values were normalized to the first group. *p* values were determined by one‐way ANOVA with Tukey's post hoc test.

To determine whether Gas6 contributes to hVIC calcification and osteogenic programming, we silenced Gas6 using siRNA (siGas6). Immunoblot analysis confirmed efficient knockdown (Figure 3F,G). Under OM conditions, siGas6 reduced RUNX2, OPN, and BMP2 protein levels (Figure 3H–J), and immunofluorescence independently showed lower RUNX2 and BMP2 signals (Figure 3K–M). Alizarin Red S staining provided a distinct mineralization readout and showed reduced OM‐induced calcium nodule formation after Gas6 knockdown (Figure 3N).

Conversely, we performed gain‐of‐function experiments using adenovirus‐mediated Gas6 overexpression (AdGas6). Successful Gas6 overexpression was validated at the protein level (Figure S3B,C). Under OM conditions, AdGas6 increased RUNX2, OPN, and BMP2 protein levels relative to AdCtrl (Figure S3B,D–F). Representative Alizarin Red S staining showed greater OM‐induced mineralization with AdGas6, with a similar qualitative trend under CM conditions (Figure S3G).

Together, these loss‐ and gain‐of‐function data support a role for Gas6 in promoting hVIC calcification and osteogenic programming.

We also examined whether Gas6 manipulation was accompanied by changes in the senescence‐associated features established in the OM‐treated hVIC model. Under OM conditions, siGas6 significantly reduced p21 and p16^INK4A^ protein levels relative to siCtrl (Figure S3H–J), while representative SA‐β‐gal images showed qualitatively less pronounced staining (Figure S3K). AdGas6 significantly increased p21 and p16^INK4A^ protein levels relative to AdCtrl under OM conditions (Figure S3L–N), accompanied by qualitatively more pronounced SA‐β‐gal staining (Figure S3O). These bidirectional changes support an association between Gas6 and senescence‐associated features in OM‐treated hVICs.

### 3.4 Gas6 Knockdown Abrogates MDV Release by Inducing a Terminal Scission Defect

Gas6 has previously been implicated in mitochondrial quality control (Kim et al. 2019). Having established its pro‐calcific role in hVICs, and given our finding that MDVs transmitted pro‐calcific activity between hVICs (Figure 1), we next examined whether Gas6 influenced MDV formation. Using the same siGas6 knockdown approach, nano‐flow cytometry analysis revealed that Gas6 knockdown reduced the total extracellular release of MDVs compared with control cells (Figure 4A,B).

**FIGURE 4:**
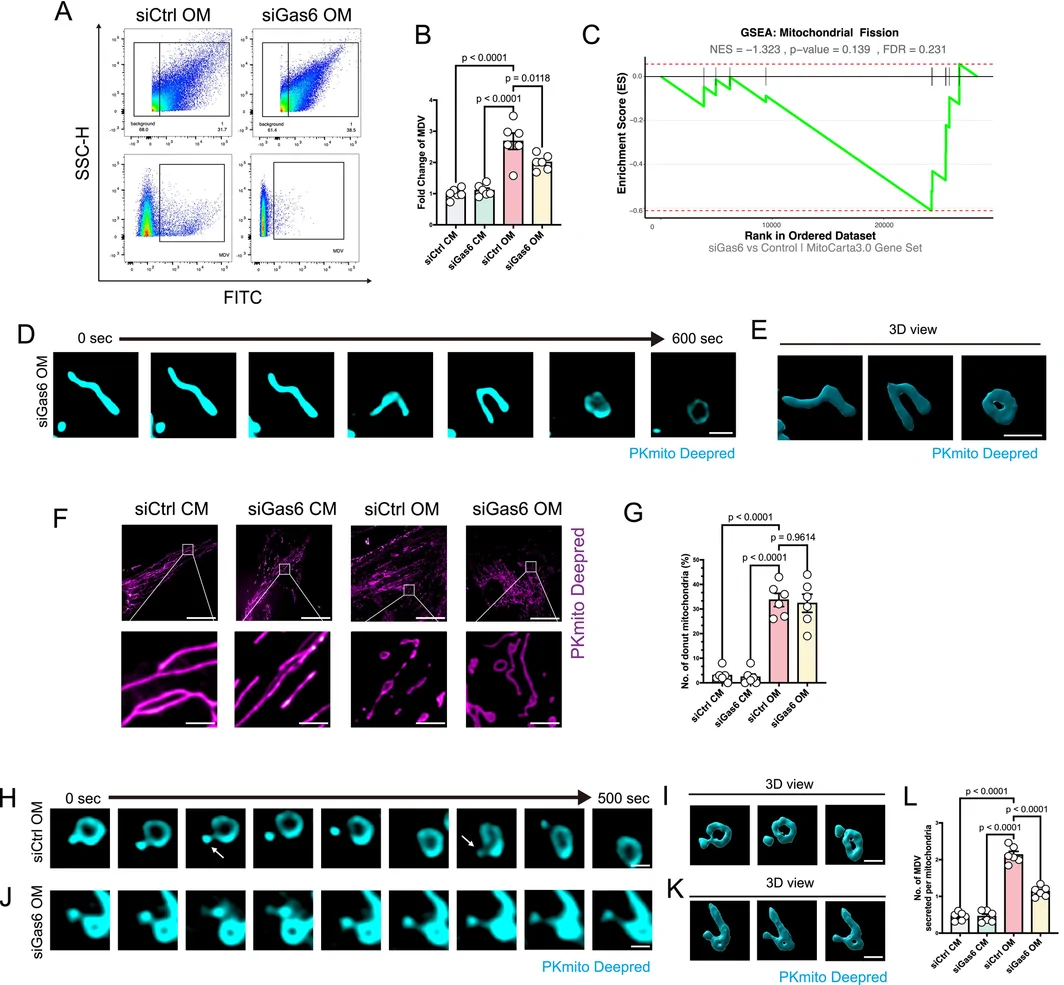
Gas6 depletion impairs MDV release by inducing a terminal scission defect. (A, B) Representative nano‐flow cytometry plots (A) and quantification (B) of MDVs in conditioned media from hVICs transfected with non‐targeting control siRNA (siCtrl) or Gas6‐targeting siRNA (siGas6) under OM conditions. (C) GSEA of the “Mitochondrial Fission” gene set from MitoCarta3.0 in siGas6 versus siCtrl hVICs under OM conditions. (D–G) Live‐cell imaging, time‐resolved three‐dimensional reconstruction, and quantification of donut‐shaped mitochondria in siCtrl and siGas6 hVICs under OM conditions. (D) Time‐lapse montage showing mitochondrial curling and self‐fusion to form donut‐shaped structures in siGas6 hVICs. Time stamps indicate seconds. Scale bar, 1 μm. See also Video S3. (E) Three selected time points from the time‐resolved three‐dimensional reconstruction of the same mitochondrial‐curling event shown in (D). The continuous reconstruction is provided in Video S4. (F) Representative live‐cell images and (G) quantification of the proportion of donut‐shaped mitochondria in siCtrl and siGas6 hVICs. Scale bars, 30 μm (overview) and 2 μm (inset). (H–L) Time‐lapse imaging, time‐resolved three‐dimensional reconstructions, and quantification of MDV budding dynamics and per‐mitochondrion MDV release. (H) Time‐lapse imaging of siCtrl OM cells showing active MDV budding and membrane detachment. See also Video S5. (I) Three selected time points from the time‐resolved three‐dimensional reconstruction of the same terminal MDV detachment/release event shown in (H). White arrows highlight the terminal budding and detachment sequence. (J) Time‐lapse imaging of siGas6 OM cells demonstrating the “stalled budding” phenotype, with nascent MDVs remaining tethered to parent mitochondria by elongated necks. See also Video S6. Scale bars in (H) and (J), 1 μm. (K) Three selected time points from the time‐resolved three‐dimensional reconstruction of the same stalled‐budding event shown in (J), with the nascent MDV remaining tethered to the parent mitochondrion at the final selected time point. The continuous reconstructions corresponding to (I) and (K) are provided side by side in Video S7. (L) Quantification of MDVs released per mitochondrion. Data are presented as mean ± SEM (*n* = 6 independent experiments). *p* values were determined by one‐way ANOVA with Tukey’s post hoc test.

Some studies have reported that MDV biogenesis may share certain components with the canonical mitochondrial fission machinery (McLelland et al. 2014; Neuspiel et al. 2008). We performed RNA sequencing to determine whether silencing Gas6 under osteogenic conditions could regulate this process. Gene set enrichment analysis (GSEA) revealed that the canonical “Mitochondrial Fission” gene signature was not significantly altered upon Gas6 knockdown (Figure 4C).

We next examined whether Gas6 is required for the structural assembly of donut‐shaped mitochondria, which serve as the physical platforms for MDV generation. Time‐lapse imaging demonstrated that mitochondria underwent spontaneous curling and self‐fusion to form donut‐shaped structures under osteogenic conditions in siGas6 hVICs (Figure 4D; Video S3). Three selected time points from the corresponding time‐resolved three‐dimensional reconstruction generated from live‐cell z‐stacks further visualized the same mitochondrial curling process (Figure 4E; continuous reconstruction in Video S4). Quantitative analysis confirmed that the percentage of donut‐shaped mitochondria remained comparable between siCtrl and siGas6 groups (Figure 4F,G), indicating that Gas6 is dispensable for formation of donut‐shaped mitochondria.

The fission transcriptional program and precursor structures were intact, yet extracellular vesicle release was reduced. This pointed to the final stage of MDV biogenesis—the budding and release process itself. We therefore performed time‐lapse imaging to directly visualize budding at the single‐organelle level. In siCtrl OM cells, we observed active MDV budding and membrane detachment (Figure 4H; Video S5). In contrast, despite possessing intact donut‐shaped precursors, siGas6 OM cells exhibited a scission defect: nascent MDVs frequently remained tethered to the parent mitochondria by elongated necks, displaying a characteristic “stalled budding” phenotype (Figure 4J; Video S6). Three selected time points from the corresponding time‐resolved three‐dimensional reconstructions generated from live‐cell z‐stacks further visualized completed terminal MDV detachment/release in siCtrl OM cells and persistent tethering in siGas6 OM cells (Figure 4I,K; continuous reconstructions in Video S7). Quantitative tracking confirmed that Gas6 knockdown significantly suppressed MDV secretion per mitochondrion (Figure 4L).

Together, these data show that Gas6 specifically controls the terminal scission of MDVs from donut‐shaped mitochondria, without affecting the fission transcriptional program or precursor formation. The “stalled budding” phenotype indicates a defect in the molecular machinery executing membrane scission.

### 3.5 Gas6 Promotes Calcification and MDV Scission Through Post‐Translational Activation of the DRP1 Machinery

Having shown that Gas6 controls both osteogenic programming (Figure 3) and MDV release (Figure 4), we next asked whether its pro‐calcific function is mediated through MDV secretion. We first revisited the “stalled budding” phenotype (Figure 4J), where Gas6‐deficient cells failed to complete the terminal scission step of MDV biogenesis. This MDV biogenesis process is orchestrated by MIRO1/2‐dependent membrane protrusion and MFF‐mediated recruitment of DRP1 for membrane fission (König et al. 2021) (Figure 5A), so we examined whether Gas6 modulates these MDV biogenesis‐related molecules. Immunoblotting revealed that the expression of MIRO1 and MIRO2 was significantly downregulated following Gas6 knockdown, whereas MFF and FIS1 expression remained unchanged (Figure 5B; Figure S5B–E). While total DRP1 protein levels remained unaffected, DRP1 phosphorylation at Ser616 was reduced, resulting in a decreased p‐DRP1/DRP1 ratio (Figure 5B; Figure S5A). This finding is consistent with our transcriptomic data showing an unaffected global fission gene program (Figure 4C). Given that the “stalled budding” phenotype is characterized by successful vesicle neck formation but failed scission, we reasoned that Gas6 is dispensable for the upstream protrusion and recruitment steps but required for DRP1 GTPase activation at the terminal scission step.

**FIGURE 5:**
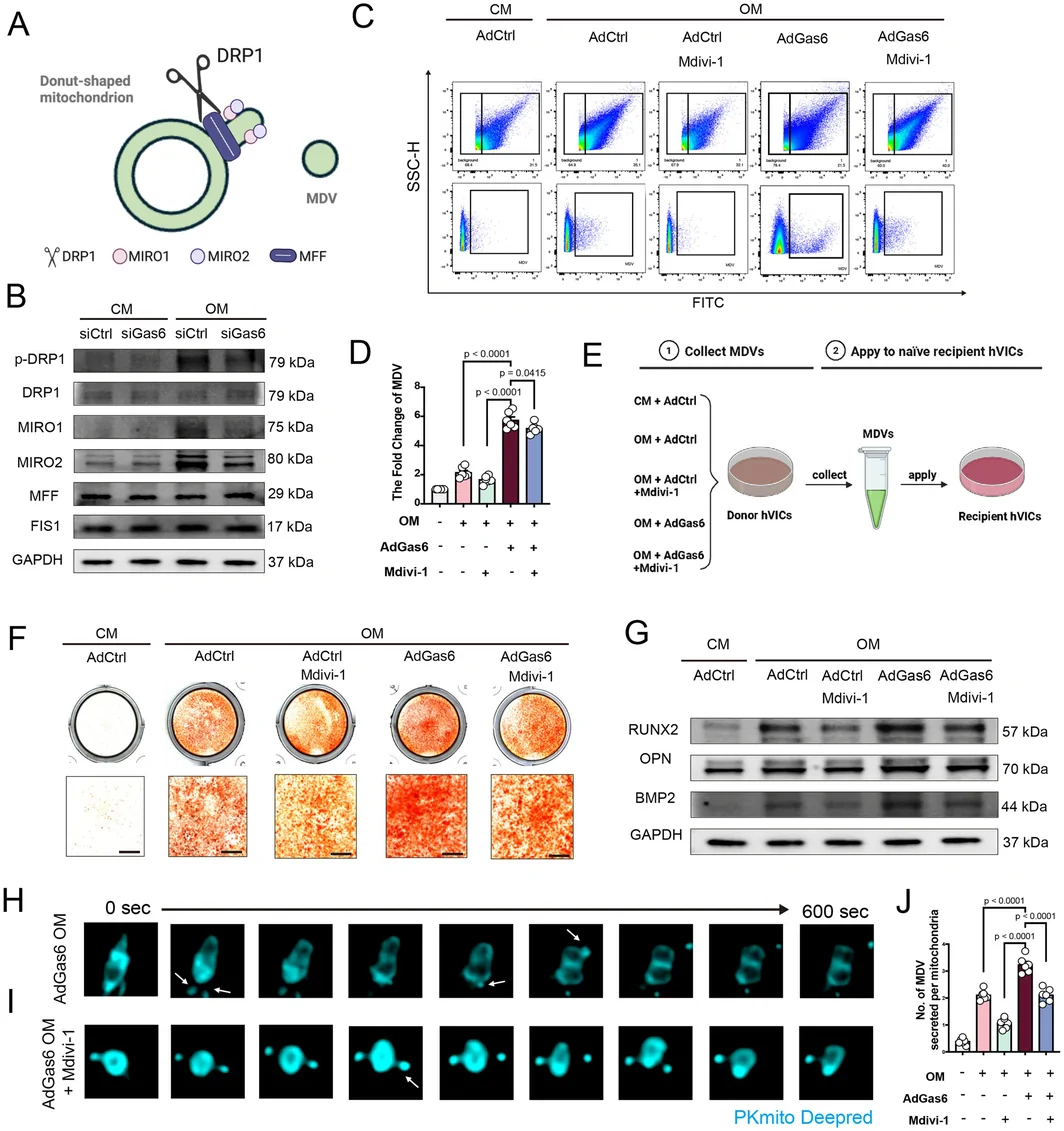
Gas6 promotes MDV scission and calcification through post‐translational activation of the dynamin‐related protein 1 (DRP1) machinery. (A) Schematic illustration of MDV biogenesis from donut‐shaped mitochondria in hVICs. MIRO1/2 facilitate membrane protrusion, MFF recruits DRP1 to the budding neck, and DRP1 executes terminal membrane scission to release the MDV. (B) Immunoblots of p‐DRP1 (Ser616), total DRP1, mitochondrial Rho GTPase 1 (MIRO1), MIRO2, mitochondrial fission factor (MFF), and fission 1 protein (FIS1) in siCtrl and siGas6 hVICs cultured under CM or OM conditions. GAPDH served as the loading control. See Figure S5A–E for densitometric quantification. (C and D) Representative nano‐flow cytometry plots (C) and quantification (D) of TOM20‐positive MDVs in conditioned media from hVICs transduced with control adenovirus (AdCtrl) or Gas6‐overexpressing adenovirus (AdGas6) under the indicated conditions. Mdivi‐1 was used to inhibit DRP1. (E) Schematic illustration of the functional MDV transfer assay. MDVs were collected from conditioned media of donor hVICs under the indicated conditions and applied to naïve recipient hVICs maintained in control medium. (F, G) Alizarin Red S staining (macroscopic and microscopic views) (F) and immunoblots of RUNX2, OPN, and BMP2 (G) in recipient hVICs following the MDV transfer assay. Scale bars, 200 μm. GAPDH served as the loading control. See Figure S5F–H for densitometric quantification. (H–J) Time‐lapse imaging of MDV budding dynamics. (H) Live‐cell imaging (PKmito Deep Red) of OM + AdGas6 cells showing active MDV budding and successful detachment. White arrows indicate successful MDV detachment in (H) and persistent tethering in (I). See also Video S8. (I) Live‐cell imaging (PKmito Deep Red) of OM + AdGas6 + Mdivi‐1 cells showing impaired vesicle detachment with nascent MDVs remaining tethered to parent mitochondria. See also Video S9. Scale bars, 1 μm. (J) Quantification of MDVs released per mitochondrion. Data are presented as mean ± SEM (*n* = 6 independent experiments). For densitometric quantification, protein expression levels were normalized to the first group. *p* values were determined by one‐way ANOVA with Tukey's post hoc test.

To selectively block the terminal scission step without disrupting Gas6 overexpression, we employed Mdivi‐1, a pharmacological inhibitor of DRP1 (Lin et al. 2025). Conditioned media from hVICs cultured under the specified conditions were collected and analyzed by nano‐flow cytometry. Gas6 overexpression (OM + AdGas6) produced the highest MDV secretion, whereas Mdivi‐1 treatment significantly blunted this hypersecretion (Figure 5C,D). Mdivi‐1 also reduced MDV release in the absence of Gas6 overexpression (OM + AdCtrl + Mdivi‐1), confirming its efficacy in blocking DRP1‐mediated scission.

To test whether differential MDV secretion translates into distinct pro‐calcific effects, we performed a functional transfer assay (Figure 5E). MDVs were isolated from equal volumes of conditioned medium from donor hVIC cultures under the indicated conditions (CM + AdCtrl, OM + AdCtrl, OM + AdCtrl + Mdivi‐1, OM + AdGas6, or OM + AdGas6 + Mdivi‐1) and applied to naïve recipient hVICs maintained in control medium. This design distinguishes the pro‐calcific effects of transferred MDV quantity from potential direct drug effects on recipient cells.

Alizarin Red S staining showed that recipient hVICs receiving MDVs from AdGas6‐overexpressing donors exhibited the strongest matrix mineralization (Figure 5F). MDVs from Mdivi‐1‐treated AdGas6 donors induced substantially less mineralization. Osteogenic markers (RUNX2, OPN, and BMP2) were highest in recipients of MDVs from the OM + AdGas6 group and significantly suppressed in the DRP1‐inhibited groups (Figure 5G; Figure S5F–H).

To directly visualize scission, we performed live‐cell imaging of mitochondrial dynamics. Mitochondria in OM + AdGas6 cells exhibited active budding events culminating in successful MDV detachment (Figure 5H; Video S8). Mdivi‐1 treatment impaired vesicle detachment; nascent MDVs remained tethered to parent mitochondria (Figure 5I; Video S9), phenocopying the “stalled budding” observed upon Gas6 knockdown. Quantitative analysis confirmed that Mdivi‐1 significantly reduced MDVs released per mitochondrion, even with concurrent Gas6 overexpression (Figure 5J).

These results show that Gas6 triggers MDV hypersecretion via post‐translational enhancement of DRP1 activity. The functional transfer assays establish MDVs as pro‐calcific mediators, and the attenuation of recipient cell calcification following DRP1‐mediated reduction of MDV secretion confirms that Gas6 exerts its pro‐calcific effects, at least partially, through MDV release.

### 3.6 A Ca^2+^/CaMKII Signaling Axis Mediates Gas6‐Induced DRP1 Activation

We identified DRP1 as the downstream effector of Gas6‐mediated MDV release, but the intracellular intermediate linking Gas6 signaling to DRP1 activation remained unclear. To address this, we re‐examined our RNA‐seq dataset. GO enrichment analysis revealed that the GO term “regulation of voltage‐gated calcium channel activity” was significantly downregulated upon Gas6 knockdown (siGas6 OM vs. siCtrl OM) (Figure 6A).

**FIGURE 6:**
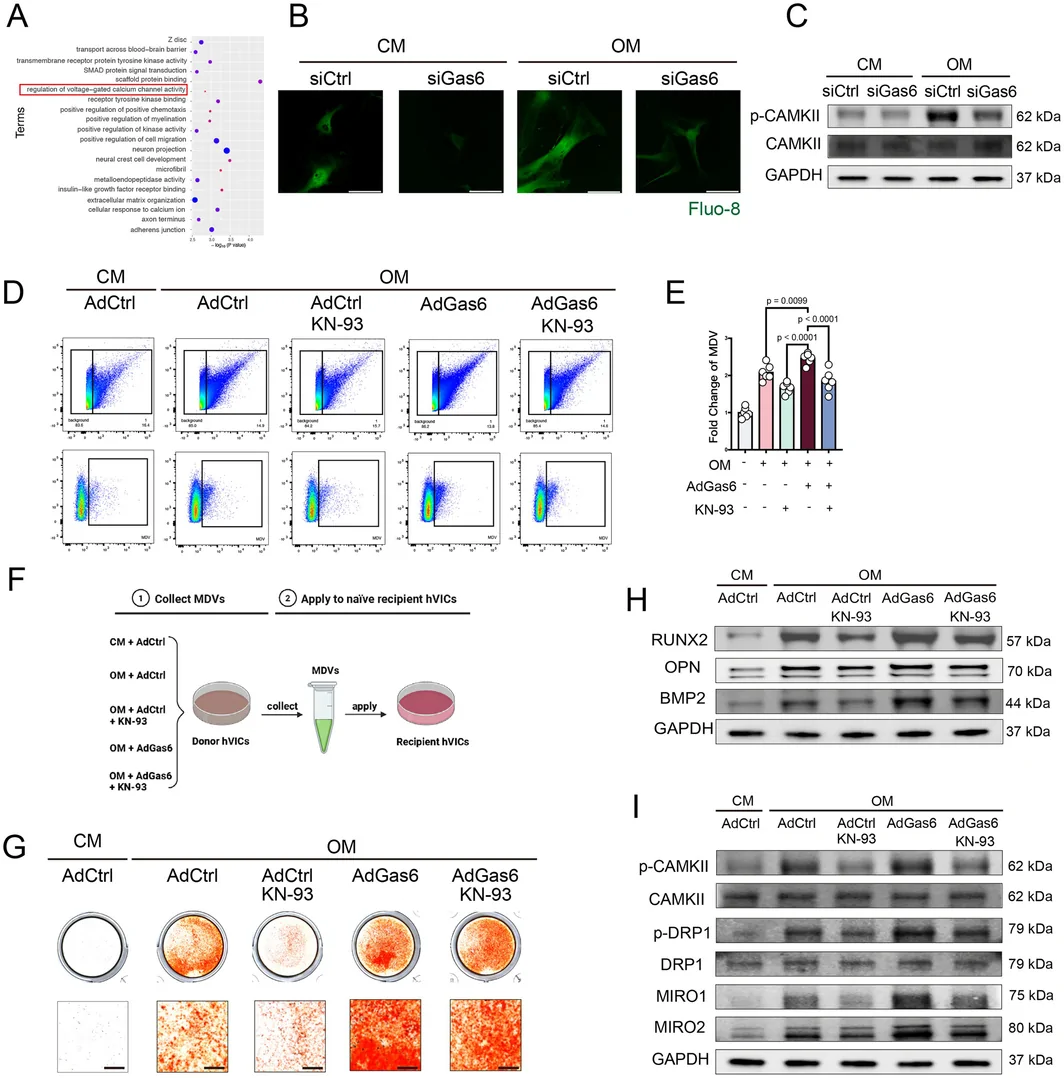
A Ca^2+^/CaMKII signaling axis mediates Gas6‐induced DRP1 activation. (A) GO enrichment analysis dot plot of RNA‐seq data from siGas6 and siCtrl hVICs under OM conditions. (B) Representative live‐cell fluorescence images of intracellular Ca^2+^ levels detected using Fluo‐8 in siCtrl and siGas6 hVICs under CM and OM conditions. Scale bars, 20 μm. See Figure S6A for quantification. (C) Representative immunoblots of p‐CaMKII and CaMKII in siCtrl and siGas6 hVICs under CM and OM conditions. GAPDH served as the loading control. See Figure S6D for quantification. (D and E) Representative nano‐flow cytometry plots (D) and quantification (E) of MDVs in conditioned media from hVICs under the indicated conditions. KN‐93 was used to selectively inhibit CaMKII. (F) Schematic illustration of the MDV functional transfer assay. (G and H) Alizarin Red S staining (G) and immunoblots of RUNX2, OPN, and BMP2 (H) in naïve recipient hVICs following the MDV transfer assay. GAPDH served as the loading control. See Figure S6G–I for densitometric quantification. Scale bars, 200 μm. (I) Immunoblots of p‐CaMKII, CaMKII, p‐DRP1, DRP1, MIRO1, and MIRO2 in AdGas6‐transduced hVICs treated with or without KN‐93. GAPDH served as the loading control. See Figure S6J for quantification. Data are presented as mean ± SEM (*n* = 6 independent experiments). For densitometric quantification, protein expression levels were normalized to the first group. *p* values were determined by one‐way ANOVA with Tukey's post hoc test.

To functionally validate this transcriptomic signature, we performed live‐cell imaging using the fluorescent calcium indicator Fluo‐8. As expected, osteogenic induction increased intracellular Ca^2+^ levels. Gas6 knockdown significantly attenuated OM‐induced Ca^2+^ influx (Figure 6B; Figure S6A). Conversely, Gas6 overexpression (OM + AdGas6) further increased intracellular Ca^2+^ accumulation compared with the OM‐treated control (Figure S6B,C).

CaMKII is a multifunctional serine/threonine kinase that transduces intracellular Ca^2+^ signals into phosphorylation‐dependent cellular responses (Swaminathan et al. 2012). Given that Gas6 knockdown downregulated calcium channel activity and attenuated Ca^2+^ influx, we hypothesized that Gas6 signals through a Ca^2+^/CaMKII axis to activate DRP1. Immunoblot analysis revealed that the p‐CaMKII/CaMKII ratio was decreased by Gas6 knockdown and elevated by Gas6 overexpression under osteogenic conditions (Figure 6C; Figure S6D–F). DRP1 inhibition with Mdivi‐1 did not alter AdGas6‐induced CaMKII phosphorylation, confirming that CaMKII operates upstream of the DRP1 scission machinery.

To determine whether Gas6 regulates MDV biogenesis via this kinase axis, we introduced KN‐93, a selective CaMKII inhibitor (Sumi et al. 1991), into our AdGas6 model. Nano‐flow cytometry showed that MDV secretion was highest in the OM + AdGas6 group and significantly reduced by KN‐93 treatment (Figure 6D,E).

To establish that CaMKII‐dependent MDV release dictates recipient cell calcification, we used a functional vesicle transfer assay (Figure 6F). MDVs isolated from equivalent volumes of conditioned medium were applied to naïve recipient cells. Naïve hVICs receiving MDVs from AdGas6‐overexpressing donor cells exhibited exacerbated calcium nodule formation. This effect was abrogated when MDVs were sourced from KN‐93‐treated donor cells (Figure 6G). Immunoblot analysis confirmed that osteogenic markers (RUNX2, OPN, and BMP2) showed a concordant stepwise reduction, being significantly suppressed after CaMKII inhibition (Figure 6H; Figure S6G–I).

Finally, we examined the molecular cascade downstream of CaMKII. KN‐93 suppressed AdGas6‐induced CaMKII phosphorylation, abolished DRP1 Ser616 phosphorylation, and reduced MIRO1 and MIRO2 expression (Figure 6I; Figure S6J). These data define a complete signaling cascade: Gas6 promotes intracellular Ca^2+^ accumulation to activate CaMKII, which phosphorylates DRP1 to drive the terminal scission and hypersecretion of pro‐calcific MDVs.

### 3.7 Gas6 Deletion Attenuates AWI‐Induced Aortic Valve Calcification and Stenosis In Vivo

Having defined the intracellular signaling axis through which Gas6 drives pro‐calcific MDV hypersecretion in vitro, we next tested its role in valvular calcification in vivo. We used a murine model of CAVD induced by aortic wire injury (AWI) (Honda et al. 2014). Wild‐type (WT) and systemic Gas6 knockout (Gas6^−/−^) mice (8–10 weeks old, both sexes, *n* = 6 per group) were subjected to either sham or AWI surgery.

Echocardiographic analysis of transvalvular haemodynamics showed that AWI induced severe aortic stenosis in WT mice (Figure 7A), characterized by elevated peak transvalvular jet velocity (Figure 7B,C) and a restricted effective orifice area (Figure 7D). Gas6 ablation (AWI Gas6^−/−^) significantly ameliorated these haemodynamic impairments, preserving the orifice area and reducing the pathological rise in jet velocity.

**FIGURE 7:**
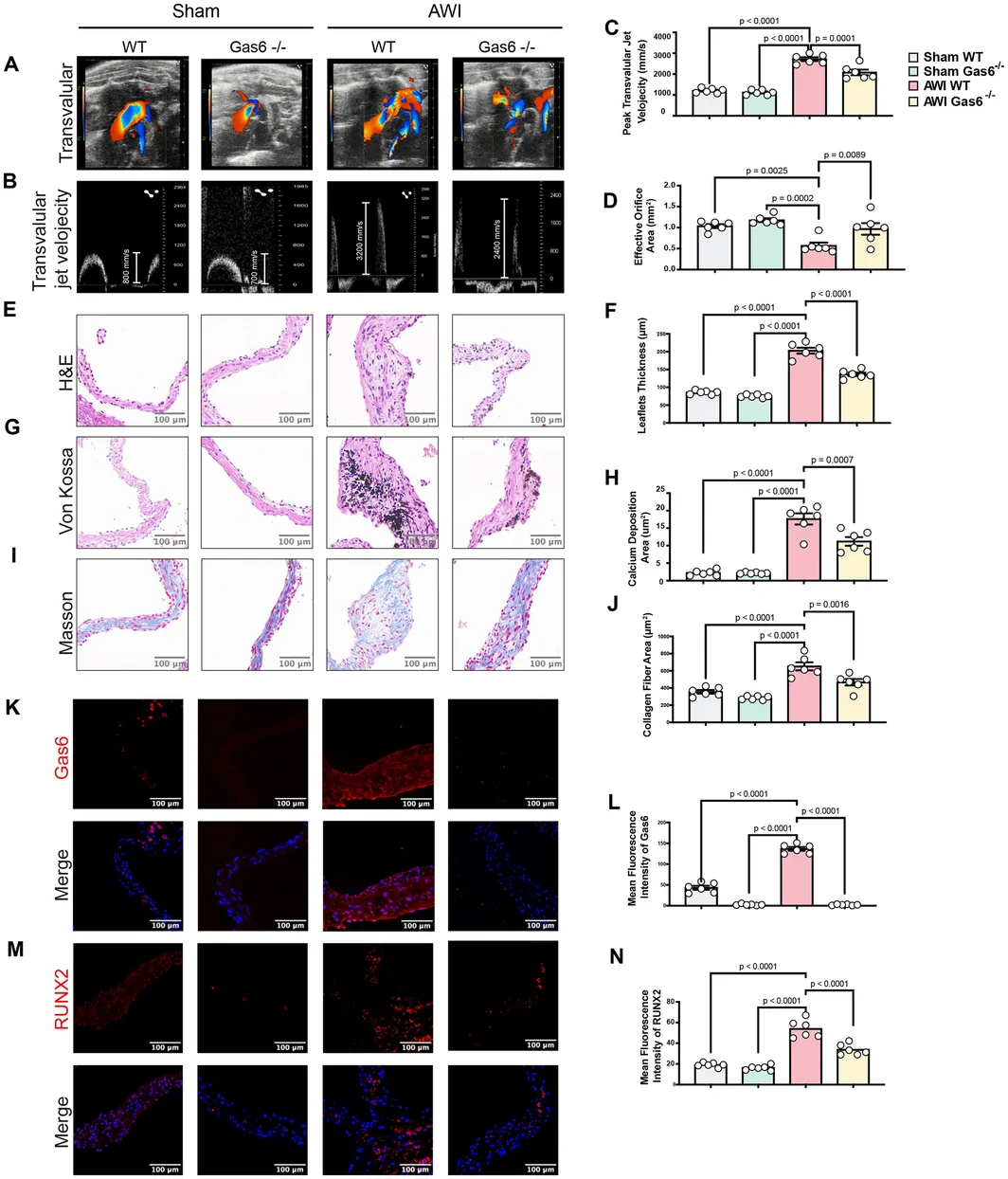
Gas6 deletion attenuates AWI‐induced aortic valve stenosis and calcification in vivo. (A–D) Echocardiographic assessment of transvalvular haemodynamics in wild‐type (WT) and Gas6^−^/^−^ mice following sham or aortic wire injury (AWI) surgery. (A) Representative color Doppler echocardiographic images of transvalvular blood flow. (B) Representative pulsed‐wave Doppler tracings of transvalvular flow velocity profiles. (C, D) Quantification of peak transvalvular jet velocity (C) and effective orifice area (D). (E–J) Histological analysis of aortic valve leaflets from WT and Gas6^−^/^−^ mice following sham or AWI surgery. (E, F) Hematoxylin and eosin (H&E) staining images (E) and quantification of leaflet thickness (F). (G, H) Von Kossa staining images (G) and quantification of calcium deposition area (H). (I, J) Modified Masson's trichrome staining images (I) and quantification of collagen fiber area (J). Scale bars, 100 μm (E, G, I). (K–N) Immunofluorescence analysis of Gas6 and RUNX2 expression in aortic valve leaflets from WT and Gas6^−/−^ mice following sham or AWI surgery. (K and L) Representative immunofluorescence images (K) and quantification of mean fluorescence intensity (L) for Gas6 (red). (M, N) Representative immunofluorescence images (M) and quantification of mean fluorescence intensity (N) for RUNX2 (red). Nuclei were counterstained with DAPI (blue). Scale bars, 100 μm. Data are presented as mean ± SEM (*n* = 6 mice per group). *p* values were determined by one‐way ANOVA with Tukey's post hoc test.

Histomorphological analysis of the murine aortic valves confirmed these findings at the tissue level. H&E staining showed that AWI‐induced leaflet thickening was attenuated in Gas6^−/−^ mice compared with WT counterparts (Figure 7E,F). Von Kossa staining revealed ectopic calcium deposition in AWI WT valve leaflets, which was significantly suppressed by Gas6 deletion (Figure 7G,H). Modified Masson's trichrome staining confirmed that collagen accumulation and fibrotic remodeling induced by AWI were significantly reduced in Gas6^−/−^ mice (Figure 7I,J).

Immunofluorescence analysis of valve leaflets showed Gas6 upregulation in AWI WT valves and its expected absence in Gas6^−/−^ cohorts (Figure 7K,L). Consistent with our in vitro findings, RUNX2 expression was elevated in AWI WT leaflets but attenuated in AWI Gas6^−/−^ mice (Figure 7M,N).

These in vivo data support a contributory role for Gas6 in AWI‐induced valvular pathology. Gas6 deletion attenuated structural valve deterioration, fibrotic remodeling, and calcification in this model.

## 4 Discussion

Our findings support a role for MDVs in the propagation of valvular calcification. MDV preparations derived from CAVD valve tissues or OM‐treated hVICs exerted pro‐calcific effects on recipient hVICs, increasing mineral deposition and osteogenic marker expression. Mechanistically, our data support a Gas6‐dependent Ca^2+^–CaMKII–DRP1 pathway that promotes terminal scission of MDVs from donut‐shaped mitochondrial precursors in calcifying hVICs. Systemic Gas6 deletion attenuated AWI‐induced aortic valve stenosis and calcification; together, these findings support the conclusion that Gas6‐regulated MDV release contributes to valvular calcification and may participate in CAVD progression.

Donut‐shaped mitochondria have been described under severe oxidative stress and energetic uncoupling (X. Liu and Hajnóczky 2011), while mitochondrial fragmentation and donut formation have been linked to mitochondrial secretion during osteogenesis in primary murine osteoblasts (Suh et al. 2023). The osteoblast findings provide a relevant precedent for donut‐associated mitochondrial secretion without establishing equivalence to the TOM20^+^/PDH^+^ vesicle population examined here. In OM‐treated primary hVICs, TOM20^+^/PDH^+^ vesicular buds were contiguous with donut‐shaped mitochondrial structures, and three‐dimensional confocal reconstruction supported their spatial continuity. TEM and three‐dimensional TEM reconstruction further supported the closed, donut‐shaped morphology of mitochondrial profiles in human CAVD valve tissue. Complementary live‐cell imaging provided temporal evidence of MDV budding and release from donut‐shaped precursors. Together, the TOM20^+^/PDH^+^ vesicle phenotype and accompanying mitochondrial remodeling are compatible with mitochondrial dysfunction under calcifying conditions, although mitochondrial function was not directly measured and MDV cargo remains undefined. To our knowledge, our study provides the first evidence of donut‐shaped mitochondria in human tissue and primary human hVICs, and identifies their functional role as platforms for pro‐calcific MDV biogenesis in CAVD.

The mitochondrial remodeling described above was observed in disease and experimental settings that also exhibited senescence‐associated features. Human CAVD valve tissues showed significantly higher p16^INK4A^ and p21 levels than nonCAVD controls, and representative SA‐β‐gal staining revealed positive foci within morphologically calcified regions; independently, OM‐treated hVICs showed significantly higher p16^INK4A^ and p21 levels and qualitatively more pronounced SA‐β‐gal staining than CM‐treated cells. Under OM conditions, Gas6 knockdown reduced and Gas6 overexpression increased p16^INK4A^ and p21 levels, with concordant qualitative changes in SA‐β‐gal staining, supporting a bidirectional association between Gas6 and these senescence‐associated features. The prior identification of GAS6 as a candidate senoprotein in a human lifespan proteomic atlas provides additional biological context for this association (Ding et al. 2025). Recipient hVICs exposed to MDV preparations derived from CAVD valve tissues or OM‐treated donor hVICs likewise showed higher p16^INK4A^ and p21 levels and qualitatively more pronounced SA‐β‐gal staining than recipients exposed to the corresponding nonCAVD‐ or CM‐derived preparations. We additionally observed that MDV release progressively increased across hVIC passages P1, P3, and P7 (Figure S1J,K). Together, these observations identify senescence‐associated features as an additional characteristic of human CAVD tissues and OM‐treated hVICs.

A key mechanistic insight from this study is the functional separation of donut‐shaped mitochondrial precursor formation from terminal MDV scission. Under OM conditions, Gas6 knockdown did not significantly alter the mitochondrial fission gene signature or the proportion of donut‐shaped mitochondria, indicating that precursor formation was preserved. In contrast, after Gas6 knockdown, nascent MDVs frequently remained tethered to parent mitochondria through elongated necks, and extracellular MDV release was reduced. Live‐cell sequences and quantitative tracking provided the principal temporal evidence for this separation; time‐resolved three‐dimensional reconstructions generated from the corresponding live‐cell z‐stacks provided complementary spatial support for completed detachment versus persistent tethering. Mechanistically, changes in intracellular Ca^2+^, CaMKII phosphorylation, and DRP1 Ser616 phosphorylation following Gas6 manipulation, together with pathway‐perturbation experiments, support a model in which the Gas6–Ca^2+^–CaMKII–DRP1 axis regulates terminal MDV scission rather than the formation of donut‐shaped precursors.

No disease‐modifying drug exists for CAVD. Our in vivo data show that systemic Gas6 deletion attenuated AWI‐induced transvalvular hemodynamic impairment and leaflet structural deterioration, supporting further investigation of Gas6 in valvular calcification. Inhibiting CaMKII with KN‐93 or DRP1 with Mdivi‐1 reduced MDV release from donor hVICs and attenuated the pro‐calcific effects of the corresponding MDV preparations on recipient hVICs in vitro, supporting CaMKII and DRP1 as functional components of this pathway. Neither protein, however, represents a straightforward therapeutic target. DRP1 is needed for normal mitochondrial fission, mitophagy, and quality control; systemic inhibition may therefore disrupt essential mitochondrial functions (Ishihara et al. 2009). CaMKII presents similar challenges. It is active in cardiac excitation–contraction coupling, neuronal plasticity, and immune signaling; these broad physiological roles may complicate systemic inhibition (Yang et al. 2003). These considerations are particularly relevant to CAVD, a chronic and slowly progressive disease. Gas6 may therefore represent a candidate upstream control point. It lies upstream of CaMKII and DRP1 and is secreted, providing a rationale for evaluating Gas6‐directed strategies.

Beyond therapy, whether MDV hypersecretion has diagnostic value remains to be determined. CAVD is currently assessed by echocardiography and CT, which identify established valvular abnormalities. Given that calcifying hVICs release TOM20‐positive vesicles into the extracellular space, future studies should determine whether circulating TOM20‐positive MDVs can be detected and whether their abundance is associated with active valvular calcification.

Several limitations should be noted. First, although our transfer experiments showed that MDV preparations exerted pro‐calcific effects, the uptake mechanisms in recipient cells and the downstream events that promote recipient‐cell calcification, such as potential activation of cGAS–STING, remain to be defined (West et al. 2015). Second, the specific molecular cargo of CAVD‐derived MDVs (e.g., oxidized mtDNA, pathogenic lipids) has not been fully characterized; doing so may reveal additional therapeutic targets. Third, Gas6‐driven MDV release in the calcifying microenvironment may represent only one mechanism through which Gas6 could promote CAVD; other mechanisms that do not depend on MDV release may also exist.

In summary, this study identifies Gas6–CaMKII–DRP1‐mediated MDV hypersecretion as a mechanism that contributes to valvular calcification and may participate in CAVD progression. These findings support further investigation of the Gas6–MDV axis as a candidate control point in valvular calcification.

## Author Contributions

M.C., F.H. conceived, designed, and supervised the experiments. Y.Z., J.Z., Y.X., and W.L. performed the experiments. F.X., J.L., S.L., X.W., and J.Z. analyzed the data and prepared figures. Z.F. obtained the study specimens and revised the manuscript. Y.Z., H.L., J.R., W.L., Z.W., and T.T. wrote the manuscript. B.S. made major contributions to confocal microscopy and three‐dimensional TEM reconstruction. All authors have read and agreed to the published version of the manuscript.

## Funding

This work was supported by the Noncommunicable Chronic Diseases‐National Science and Technology Major Project (2026ZD0554300), the National Natural Science Foundation of China (U23A20395, 82170375, 82500455, 82600733), the Sichuan Provincial Natural Science Foundation (2026NSFSC0552), the Fundamental Research Funds for the Central Universities, the Science and Technology Projects of Xizang Autonomous Region, China (XZ202501ZY0120), and the “1.3•5” project of West China Hospital (GFXK000010401), Sichuan University (ZYGD 23021, 23HXFH009).

## Conflicts of Interest

The authors declare no conflicts of interest.

## Supporting information


**Data S1:** Unedited immunoblots.

**Supporting Information S1:** Supplementary methods, figures, and tables.
**Figure S1:** Osteogenic stimulation enhances MDV biogenesis and characteristic mitochondrial morphological remodeling, related to Figure 1.
**Figure S2:** Senescence‐associated marker changes in MDV‐exposed recipient hVICs and Gas6 expression in HFD‐induced murine valves, related to Figure 2.
**Figure S3:** Effects of Gas6 manipulation on hVIC calcification, osteogenic programming, and senescence‐associated features, related to Figure 3.
**Figure S5:** Densitometric quantification of DRP1 scission machinery components in siCtrl and siGas6 hVICs and osteogenic marker expression in recipient hVICs, related to Figure 5.
**Figure S6:** Quantitative and gain‐of‐function validation of the Gas6–Ca^2+^–CaMKII axis, related to Figure 6.
**Table S1:** Clinical characteristics of patients providing human aortic valve tissue.
**Table S2:** Primary antibodies for immunoblotting.
**Table S3:** Antibodies for immunofluorescence staining.
**Table S4:** Antibodies for nano‐flow cytometry.
**Table S5:** Primer sequences for Gas6 mouse genotyping PCR.
**Table S6:** siRNA Sequences.

**Video S1:** Rotational view of a three‐dimensional reconstruction from confocal z‐stacks, related to Figure 1N. Rotational view of the static three‐dimensional reconstruction of a TOM20^+^/PDH^+^ donut‐shaped mitochondrial structure with an attached vesicular bud in an OM‐treated hVIC. Rotation of the reconstructed volume is shown for spatial visualization and does not represent time‐lapse imaging.

**Video S2:** Multi‐angle rotational view of a three‐dimensional TEM reconstruction, related to Figure 1O. Multi‐angle rotational view of the static three‐dimensional reconstruction corresponding to the donut‐shaped mitochondrial profile in human CAVD valve tissue shown in Figure 1O. Rotation of the reconstructed volume is shown for spatial visualization and does not represent time‐lapse imaging.

**Video S3:** Gas6 depletion does not impair formation of donut‐shaped mitochondria, related to Figure 4D. Time‐lapse imaging of mitochondria (PKmito Deep Red) in siGas6 hVICs under OM conditions. Despite Gas6 knockdown, elongated mitochondria undergo spontaneous curling and self‐fusion to form closed donut‐shaped structures. Images were acquired at 25‐s intervals. Scale bar, 1 μm.

**Video S4:** Time‐resolved three‐dimensional reconstruction of mitochondrial curling after Gas6 depletion, related to Figure 4E. The continuous reconstruction was generated from sequential confocal z‐stacks acquired at each time point for the same mitochondrial‐curling event shown in Figure 4D; Figure 4E shows three selected time points from this reconstruction.

**Video S5:** Active MDV budding and release in control cells, related to Figure 4H. Time‐lapse imaging of mitochondria (PKmito Deep Red) in siCtrl hVICs under OM conditions, demonstrating active MDV budding from donut‐shaped mitochondria with successful membrane detachment and vesicle release. Images were acquired at 25‐s intervals. Scale bar, 1 μm.

**Video S6:** “Stalled budding” phenotype in Gas6‐depleted cells, related to Figure 4J. Time‐lapse imaging of mitochondria (PKmito Deep Red) in siGas6 hVICs under OM conditions. Despite the presence of intact donut‐shaped precursors, nascent MDVs remain persistently tethered to parent mitochondria by elongated membrane necks without completing membrane scission. Images were acquired at 25‐s intervals. Scale bar, 1 μm.

**Video S7:** Time‐resolved three‐dimensional comparison of MDV detachment and persistent tethering, related to Figure 4I,K. The continuous reconstructions were generated from sequential confocal z‐stacks acquired at each time point for the same events shown in Figure 4H,J and are presented side by side. In siCtrl hVICs under OM conditions, the nascent MDV completed terminal detachment/release; in siGas6 hVICs under OM conditions, the nascent MDV remained persistently tethered to the parent mitochondrion without completing membrane scission. Figure 4I,K each show three selected time points from the corresponding reconstruction.

**Video S8:** Active MDV budding and release in Gas6‐overexpressing cells, related to Figure 5. Time‐lapse imaging of mitochondria (PKmito Deep Red) in AdGas6‐transduced hVICs under OM conditions. Donut‐shaped mitochondria exhibit active MDV budding events culminating in successful membrane scission and vesicle release. Images were acquired at 25‐s intervals. Scale bar, 1 μm.

**Video S9:** DRP1 inhibition impairs MDV detachment in Gas6‐overexpressing cells, related to Figure 5. Time‐lapse imaging of mitochondria (PKmito Deep Red) in AdGas6‐transduced hVICs under OM conditions treated with Mdivi‐1. Nascent MDVs remain persistently tethered to parent mitochondria without completing membrane scission, phenocopying the “stalled budding” observed upon Gas6 knockdown. Images were acquired at 25‐s intervals. Scale bar, 1 μm.

## Data Availability

The datasets generated and analyzed during the current study are available from the corresponding author upon reasonable request.
