# Supplementary material for: The Senokine Gas6 Promotes Aortic Valve Calcification Through CaMKII–DRP1–Mediated Terminal Scission of Mitochondrial‐Derived Vesicles
Source: Aging Cell. 2026 Oct 20;25(11):e70758. doi: 10.1111/acel.70758 (PMC13684317; doi:10.1111/acel.70758)
Supplement: Supplementary file 1 [file ACEL-25-e70758-s003.docx]

**SUPPLEMENTARY MATERIALS**

**——** UNEDITED WESTERN BLOTS

**The senokine Gas6 promotes aortic valve calcification through CaMKII–DRP1–mediated terminal scission of mitochondrial-derived vesicles**

**Authors**

Yaoyu Zhang^1,2,3#^, Jialiang Zhang^1,2,3#^, Yanjiani Xu^1,2,3#^, Wenhua Lei^1,2,3#^, Fei Xu^1,2,3^, Junli Li^1,2,3^, Zisong Wei^1,2,3^, Bo Su^5^, Shuang Liu^1,2,3^, Jing Zhou^1,2,3^, Hongde Li^1,2,3^, Junpeng Ran^1,2,3^, Weiya Li^1,2,3^, Tuerhongjiang Tuerdi^1,2,3^, Xiaojiao Wang^5^, Zhi Fang^4^*, Fangyang Huang^1,2,3^*, Mao Chen^1,2,3^*

**Affiliations**

1.Laboratory of Cardiac Structure and Function, Institute of Cardiovascular Diseases, West China Hospital, Sichuan University, Chengdu, 610041, PR China.

2.Department of Cardiology, West China Hospital, Sichuan University, No.37 Guoxue Street, Chengdu 610041, P.R. China

3.Cardiac Structure and Function Research Key Laboratory of Sichuan Province, West China Hospital, Sichuan University, Chengdu, 610041, PR China

4. Department of Cardiovascular Surgery, West China Hospital, Sichuan University

5.Core Facilities of West China Hospital, Chengdu, Sichuan, China

\# These authors contributed equally to this work.

*Correspondence to Mao Chen (hmaochen@vip.sina.com), Fangyang Huang (fyhuang1989@wchscu.cn), Zhi Fang (fangzhi@wchscu.cn)

**Content of this document.**

UNEDITED WESTERN BLOTS

**Left panels show the original, unprocessed fluorescent Western blot images as directly exported from the imaging system. Due to the limited visibility of fluorescent bands, color-inverted and appropriately cropped versions are provided on the right panels for clearer visualization. No additional image manipulation was performed.**

**Figure 1B.** Immunoblot of EV markers (CD9, CD63), mitochondrial outer membrane protein TOM20, and cytoplasmic marker β-actin (negative control) in EVs isolated from the perfusate of nonCAVD and CAVD valve leaflets.

**Figure 1H.** Immunoblot of RUNX2, OPN, and BMP2 in recipient hVICs treated with MDV^nonCAVD^ or MDV^CAVD^.

**Figure 1L.** Immunoblot analysis of RUNX2, OPN, and BMP2 in recipient hVICs treated with MDV*^CM^* or MDV*^OM^*.

**Figure 2B. Immunoblots** of p21 and p16*^INK4A^* protein expression in nonCAVD and CAVD valve tissues.

**Figure 2H.** Immunoblots of p21 and p16*^INK4A^* protein expression in hVICs cultured in control medium (CM) or osteogenic medium (OM).

**Figure 2M. Immunoblots** of Gas6 and RUNX2 protein expression in nonCAVD and CAVD valve tissues.

(The Western blots shown in Figure 2M were performed in the initial phase of this study using HRP-conjugated secondary antibodies and chemiluminescence detection, whereas all subsequent Western blots in this manuscript were performed using fluorescent secondary antibodies. The original chemiluminescence films/images for Figure 2M are provided below.)

**Figure 3A.** Immunoblots of Gas6, RUNX2, BMP2, and OPN in hVICs cultured in control medium (CM) or osteogenic medium (OM) for 3, 7, and 14 days.

**Figure 3F.** Immunoblots of Gas6, RUNX2, OPN, and BMP2. hVICs treated with **siCtrl (non-targeting control siRNA) or siGas6 (Gas6-targeting siRNA)** under CM and OM conditions.

**Figure S3B.** Immunoblots of Gas6, RUNX2, OPN, and BMP2 in AdCtrl- and AdGas6-transduced hVICs under CM and OM conditions.

**Figure S3H.** Immunoblots of p21 and p16*^INK4A^* in siCtrl- and siGas6-transduced hVICs under CM and OM conditions.

**Figure S3L.** Immunoblots of p21 and p16*^INK4A^* in AdCtrl- and AdGas6-transduced hVICs under CM and OM conditions.

**Figure 5B.** Immunoblots of p-DRP1 (Ser616), total DRP1, MIRO1, MIRO2, MFF, and FIS1 in siCtrl and siGas6 hVICs cultured under CM or OM conditions.

**Figure 5G.** immunoblots of OPN, RUNX2, and BMP2 in recipient hVICs following the MDV transfer assay.

**Figure 6C.** Immunoblots of p-CaMKII and CaMKII in siCtrl and siGas6 hVICs under CM and OM conditions.

**Figure 6H.** Immunoblots of OPN, RUNX2, and BMP2 in naïve recipient hVICs following the MDV transfer assay.

**Figure 6I.** Immunoblots of p-CaMKII, CaMKII, p-DRP1, DRP1, MIRO1 and MIRO2 in AdGas6-transduced hVICs treated with or without KN-93.

**Figure S6E.** Immunoblots of the p-CaMKII/CaMKII in AdGas6-transduced hVICs under OM conditions treated with or without Mdivi-1.
