# Supplementary material for: The Senokine Gas6 Promotes Aortic Valve Calcification Through CaMKII–DRP1–Mediated Terminal Scission of Mitochondrial‐Derived Vesicles
Source: Aging Cell. 2026 Oct 20;25(11):e70758. doi: 10.1111/acel.70758 (PMC13684317; doi:10.1111/acel.70758)
Supplement: Supplementary file 2 [file ACEL-25-e70758-s008.docx]

**SUPPLEMENTARY MATERIALS**

**The senokine Gas6 promotes aortic valve calcification through CaMKII–DRP1–mediated terminal scission of mitochondrial-derived vesicles**

**Authors**

Yaoyu Zhang^1,2,3#^, Jialiang Zhang^1,2,3#^, Yanjiani Xu^1,2,3#^, Wenhua Lei^1,2,3#^, Fei Xu^1,2,3^, Junli Li^1,2,3^, Zisong Wei^1,2,3^, Bo Su^5^, Shuang Liu^1,2,3^, Jing Zhou^1,2,3^, Hongde Li^1,2,3^, Junpeng Ran^1,2,3^, Weiya Li^1,2,3^, Tuerhongjiang Tuerdi^1,2,3^, Xiaojiao Wang^5^, Zhi Fang^4^*, Fangyang Huang^1,2,3^*, Mao Chen^1,2,3^*

**Affiliations**

1. Laboratory of Cardiac Structure and Function, Institute of Cardiovascular Diseases, West China Hospital, Sichuan University, Chengdu, 610041, PR China.

2. Department of Cardiology, West China Hospital, Sichuan University, No. 37 Guoxue Street, Chengdu 610041, PR China.

3. Cardiac Structure and Function Research Key Laboratory of Sichuan Province, West China Hospital, Sichuan University, Chengdu, 610041, PR China.

4. Department of Cardiovascular Surgery, West China Hospital, Sichuan University.

5. Core Facilities of West China Hospital, Chengdu, Sichuan, China.

\# These authors contributed equally to this work.

*Correspondence to Mao Chen (hmaochen@vip.sina.com), Fangyang Huang (fyhuang1989@wchscu.cn), Zhi Fang (fangzhi@wchscu.cn)

**Supplementary Materials**

**This file includes:**

**Supplementary Text**

**Supplementary Figures**

**Supplementary Videos**

**Supplementary Tables**

**Supplementary Text**

**Methods**

**Human Samples**

Calcified aortic valve leaflets were obtained from patients undergoing surgical aortic valve replacement for severe calcific aortic valve disease (CAVD). Non-calcified control tissues (nonCAVD) were harvested from age- and sex-matched patients undergoing cardiac surgery for pure aortic regurgitation without calcification. The cohort comprised 24 patients aged 60–73 years, including 12 nonCAVD controls and 12 patients with CAVD. The mean ages of the nonCAVD and CAVD groups were 64.4 ± 2.6 and 65.4 ± 4.0 years, respectively (mean ± SD; P = 0.474). Six patients per group provided valve tissue for ex vivo hemodynamic perfusion and tissue-derived mitochondrial-derived vesicle (MDV) experiments. The remaining six patients per group provided tissue for human histological and molecular analyses, including assessments of senescence-associated features. Primary human valvular interstitial cells (hVICs) used in all in vitro experiments were isolated exclusively from the six nonCAVD donors in the second patient subset. Calcified and non-calcified regions within each CAVD valve leaflet were identified based on preoperative computed tomography (CT) and distinguished primarily by gross visual inspection, with stereomicroscopic examination used when necessary. Regions containing calcific nodules were classified as calcified, whereas regions with normal leaflet thickness and no apparent calcification were classified as non-calcified. This study was conducted in strict accordance with the ethical guidelines set forth in the Declaration of Helsinki and was approved by the Ethics Committee of West China Hospital (Approval No. 2022-1874). Written informed consent was obtained from all participants. Clinical characteristics of the study participants are presented in Table S1.

**Primary Human Valvular Interstitial Cell Isolation and Culture**

Aortic valves were washed three times in phosphate-buffered saline (PBS) and subjected to enzymatic digestion in 1 mg/mL type II collagenase (Gibco, 17101-015) for four hours at 37 °C in a humidified atmosphere containing 5% CO₂. The cell suspension was filtered through a 70 μm cell strainer (Biosharp, 697588) and centrifuged at 300 × g for ten minutes. Isolated hVICs were resuspended and cultured in high-glucose Dulbecco's modified Eagle's medium (DMEM, Gibco, 6125074) supplemented with 10% fetal bovine serum (FBS, Biochannel, BC-SE-FBS01) and 1% penicillin/streptomycin (Gibco, 15140122) at 37 °C in a humidified atmosphere containing 5% CO₂. The medium was replaced every 2–3 days. Cells between passages three and six were used for all experiments unless otherwise indicated; the passage-comparison experiment used hVICs at P1, P3, and P7.

**Pro-Osteogenic Medium Treatment**

hVICs were cultured in pro-osteogenic medium (OM) consisting of DMEM supplemented with 5% FBS, 10 mM β-glycerophosphate (Sigma-Aldrich, G5422-100G), 50 μg/mL ascorbic acid (Sigma-Aldrich, 49752-10G), and 10 nM dexamethasone (Sigma-Aldrich, D4902). Control cells were maintained in standard control medium (CM) consisting of DMEM supplemented with 5% FBS alone. Except for the time-course experiment, in which hVICs were analyzed after 3, 7, and 14 days, cells in all other CM and OM experiments were cultured for 21 days. The medium was replaced every 2–3 days. For dose-dependent experiments, hVICs were exposed to 0.5×, 1.0×, or 2.0× concentrations of the OM while maintaining a constant 5% FBS concentration.

**Ex Vivo Hemodynamic Simulation System**

Freshly harvested aortic valve leaflets (processed within two hours of excision) were mounted in a custom pulsatile flow chamber connected to a closed-loop peristaltic pump system. Equal wet weights of valve tissue were used across all experiments. The perfusion circuit was filled with serum-free DMEM supplemented with 5% penicillin–streptomycin and maintained at 37 °C in a humidified incubator with 5% CO₂. Pulsatile flow was applied at 1 Hz to simulate physiological cardiac cycling. Wall shear stress acting on the leaflet surface was estimated using a parallel-plate approximation (τ = 6μQ/wh²; w = 0.5 cm, h = 2 mm), yielding physiological shear stress levels of approximately 10–15 dyn/cm². After 6 hours of continuous pulsatile perfusion, the circulating medium was collected and subjected to differential ultracentrifugation as described below to isolate the total extracellular vesicle fraction, which was resuspended in sterile PBS for downstream analyses.

**Isolation of Extracellular Vesicles**

Extracellular vesicles (EVs) were isolated from conditioned medium or ex vivo perfusate using differential ultracentrifugation. Conditioned medium (10 mL per condition) was collected and centrifuged sequentially at 200 × g for five minutes at 4 °C and at 2,000 × g for 10 minutes at 4 °C to remove cells and debris. The supernatant was filtered through a 0.22 μm membrane filter (Millipore, IPVH00010) and subjected to ultracentrifugation at 100,000 × g for 70 minutes at 4 °C (Beckman Coulter, Optima XPN-100, SW41 rotor). The EV pellet was resuspended in PBS for downstream analyses.

**Nanoparticle Tracking Analysis**

The size distribution and concentration of isolated vesicles were characterized using nanoparticle tracking analysis (NTA; ZetaView, Particle Metrix, Germany). Samples were diluted in PBS to achieve an optimal particle concentration of 10⁷–10⁸ particles/mL and analyzed using ZetaView software. Three independent measurements were performed for each sample, and mean values were reported.

**Nano-Flow Cytometry**

Nano-flow cytometry analysis was performed using a **BD FACSDiscover S8 spectral cell sorter** (BD Biosciences) equipped with a 488 nm laser. Gating was established based on apparent particle size (50–500 nm) using silica nanoparticle size standards (COSMO BIO, Megamix-Plus SSC); the wider gate accounts for the fluorescence-induced apparent size shift of antibody-labeled vesicles in the software detection algorithm. Isolated EVs were incubated with FITC-conjugated anti-TOM20 antibody (ABclonal, A26783) in 50 μL PBS for 90 minutes at room temperature in the dark. Antibody aggregates were removed by centrifugation at 12,000 × g for ten minutes. Stained EVs were collected by ultracentrifugation at 100,000 × g for 70 minutes at 4 °C and resuspended in PBS. TOM20-positive mitochondrial-derived vesicles (MDVs) were identified as events within the size gate exhibiting fluorescence intensity above the isotype control threshold. Isotype-matched IgG served as the negative control.

**MDV Functional Transfer Assay**

Purified MDVs from donor hVICs cultured under the indicated conditions were resuspended in serum-free medium and applied to naïve recipient hVICs maintained in control medium. Recipient cells were cultured for 21 days with medium replacement every three days, after which matrix mineralization and osteogenic marker expression were assessed by Alizarin Red S staining and immunoblot analysis, respectively.

**Purification of Mitochondrial-Derived Vesicles by Immunoprecipitation**

For functional studies requiring enriched MDVs, an immunoprecipitation-based isolation strategy was employed. Magnetic beads (MCE, HY-K0222) were prewashed with PBS and incubated with anti-TOM20 antibody (ABclonal, A26783). After washing to remove unbound antibodies, the antibody-conjugated beads were incubated with the crude EV preparation overnight at 4 °C. Bead-bound MDVs were collected using a magnetic stand and eluted with 0.1 M glycine buffer (pH 2.8) for ten minutes at room temperature, followed by immediate neutralization with 1 M Tris-HCl (pH 9.0). Eluted MDVs were resuspended in serum-free medium for functional transfer assays or stored at −80 °C.

**Immunoblot Analysis**

Proteins were extracted using RIPA buffer (Thermo Fisher Scientific, 89900) supplemented with protease and phosphatase inhibitors (Beyotime, P1005) and quantified using a BCA assay kit (Beyotime, P0012). Equal amounts of protein were resolved on 10% SDS-PAGE gels and transferred onto PVDF membranes (Millipore, IPVH00010). Membranes were blocked with 5% nonfat dry milk in Tris-buffered saline with 0.5% Tween 20 (TBS-T), incubated overnight at 4 °C with primary antibodies (Table S2), and then with HRP-conjugated secondary antibodies for one hour at room temperature. Bands were visualized using enhanced chemiluminescence (Beyotime, P0018S) and quantified using ImageJ. GAPDH or β-actin served as loading controls.

**Transmission Electron Microscopy**

For cellular ultrastructural analysis, hVICs cultured under CM or OM conditions were fixed with 2.5% glutaraldehyde at 4 °C for eight hours, post-fixed with 1% osmium tetroxide for two hours, dehydrated through a graded acetone series, and embedded in Araldite resin. Ultrathin sections (70 nm) were cut using an ultramicrotome, counterstained with uranyl acetate and lead citrate, and examined by TEM. Clinical valve specimens from CAVD and nonCAVD patients were processed identically to examine mitochondrial morphology in native valve tissue. All TEM images were acquired on a transmission electron microscope (HT7700, Hitachi, Japan) operated at 80 kV. Three-dimensional TEM reconstructions were generated from five consecutive ultrathin sections (70 nm per section) using Imaris software (version 10.1.1).

**Live-Cell Imaging and Single-Organelle Tracking**

hVICs were seeded in glass-bottom dishes (Cellvis, D35-20-1.5-N) and stained with PKmito Deep Red (Spirochrome) according to the manufacturer's instructions. Time-lapse imaging was performed on a laser scanning confocal microscope (LSM980, Zeiss) equipped with a temperature-controlled stage maintained at 37 °C with 5% CO₂. Images were acquired at 25-second intervals for 9 to 10 minutes using a 63× oil immersion objective. Time-resolved three-dimensional reconstructions were generated from sequential confocal z-stacks acquired at each time point using Imaris software (version 10.1.1).

For quantitative image analysis, donut-shaped mitochondria were defined as toroidal structures with a clearly visible central lumen devoid of PKmito Deep Red fluorescence. Classification was performed independently by two observers blinded to the experimental conditions, with a minimum of 20 mitochondria per cell and 10 cells per condition analyzed. The percentage of donut-shaped mitochondria was calculated as the number of toroidal structures divided by the total number of mitochondria counted per cell. MDV budding events were defined as discrete vesicular structures observed to separate from parent mitochondria in consecutive time-lapse frames and quantified manually. The number of released MDVs per mitochondrion was calculated from time-lapse sequences.

**Isobaric Tags for Relative and Absolute Quantitation (iTRAQ) Proteomics of Human Valve Tissues**

iTRAQ proteomic data from human CAVD and nonCAVD valve tissues were obtained from our previously published dataset (Shao et al., 2026). Protein extraction, peptide labeling, fractionation, liquid chromatography-tandem mass spectrometry analysis, protein identification and quantification, and differential-expression analysis were performed as described previously.

**Bulk RNA Transcriptomics of Human Valve Tissues**

Bulk RNA transcriptomic data from human CAVD and nonCAVD valve tissues were obtained from the same previously published dataset (Shao et al., 2026). Differentially expressed genes and proteins were integrated to identify candidates concordantly upregulated at the transcript and protein levels. Overlapping candidates were visualized as a Venn diagram (related to Figure 2L).

**Small Interfering RNA-Mediated Gene Silencing**

siRNAs targeting Gas6 (siGas6) and non-targeting negative control siRNA (siCtrl) were designed and synthesized by Genepharma. The siRNA sequences are provided in Table S6. hVICs were seeded at 60–70% confluence and allowed to adhere for 24 hours. Transfection was performed using Lipofectamine RNAiMAX (Thermo Fisher Scientific, 13778150). Cells were cultured for six hours post-transfection to allow siRNA-mediated gene silencing. Knockdown efficiency was confirmed by immunoblot analysis prior to subsequent experimental procedures.

**Adenovirus-Mediated Gene Overexpression**

Recombinant adenoviral vectors encoding human Gas6 (AdGas6) and control adenovirus (AdCtrl) were procured from WZ Biosciences. hVICs were transduced with adenovirus at a multiplicity of infection (MOI) of 25. Following a 24-hour incubation period, overexpression efficiency was confirmed by immunoblot analysis, and cells were then subjected to the indicated experimental interventions.

**RNA Sequencing of hVICs and Bioinformatic Analysis**

Total RNA was extracted from siCtrl and siGas6 hVICs cultured under OM conditions using the Total RNA Isolation Kit (Vazyme, RC112-01). RNA integrity was evaluated using an Agilent Bioanalyzer. cDNA libraries were prepared using the TruSeq RNA Sample Preparation Kit (Illumina) and sequenced on an Illumina NovaSeq 6000 platform with paired-end 150 bp reads. Raw sequencing reads were quality-controlled and filtered using fastp (v0.23.0), with removal of adapter sequences, reads shorter than 15 bp, low-quality reads (>8% bases below Q20), and reads containing more than five ambiguous bases (N). Filtered reads were mapped to the human reference genome (GRCh38) using STAR (v2.7.6a), and gene expression levels were quantified as reads per kilobase per million mapped reads (RPKM). Differentially expressed genes (DEGs) were identified using edgeR (v3.40.2), with significance defined as |log2FC| > 1 and P < 0.05.

Gene set enrichment analysis (GSEA) was performed using GSEA software (Broad Institute, v4.1.0) with the MitoCarta3.0 gene set applied to the ranked gene list from siGas6 versus siCtrl hVICs under OM conditions. Gene sets with a false discovery rate (FDR) < 0.25 and nominal *P* < 0.05 were considered significantly enriched (related to Figure 4C).

Gene Ontology (GO) enrichment analysis of DEGs from the same siGas6 versus siCtrl comparison was performed using clusterProfiler (v4.0) in R. GO terms from the Biological Process ontology were tested for overrepresentation using a hypergeometric test. Enriched GO terms were filtered by adjusted *P* < 0.05 and visualized as dot plots using ggplot2 (related to Figure 6A).

**Pharmacological Inhibitors**

To inhibit DRP1-mediated mitochondrial scission, hVICs were treated with Mdivi-1 (10 μM, MCE, HY-15886). To inhibit CaMKII, hVICs were treated with KN-93 (10 μM, MCE, HY-15465). An equivalent volume of dimethyl sulfoxide (DMSO, MCE, HY-Y0320) served as the vehicle control for all inhibitor experiments.

**Alizarin Red S Staining**

Calcium deposition was assessed by Alizarin Red S staining. hVICs cultured in 12-well or 24-well plates were washed twice with PBS and fixed with 4% paraformaldehyde for 15 minutes at room temperature. After washing with distilled water, cells were stained with 2% Alizarin Red S solution (Cyagen Biosciences, ALIR-10001) for 30 minutes at room temperature. Excess stain was removed by repeated washing with distilled water. Images were obtained using an inverted microscope (Olympus, DMI8). The stained area was quantified using ImageJ software.

**Senescence-Associated β-Galactosidase Staining**

Frozen human valve sections and cultured hVICs were stained for senescence-associated β-galactosidase (SA-β-gal) activity using a Senescence β-Galactosidase Staining Kit (Beyotime, C0602) according to the manufacturer’s instructions.

**Intracellular Calcium Imaging**

Intracellular calcium levels were assessed using the fluorescent calcium indicator Fluo-8 AM (Abcam, ab112129). hVICs were incubated with 5 μM Fluo-8 AM in Hanks' balanced salt solution (HBSS) for 30 minutes at 37 °C in the dark. After washing twice with HBSS, live-cell imaging was performed on a laser scanning confocal microscope (LSM980, Zeiss) with excitation at 488 nm and emission collected at 500–550 nm. Mean fluorescence intensity was quantified using ImageJ software.

**Histological Staining of Valve Tissues**

Valve specimens were fixed in 4% paraformaldehyde overnight at 4 °C, dehydrated through a graded ethanol series, embedded in paraffin, and sectioned at 5 μm. For frozen sections, tissues were embedded in optimal cutting temperature (OCT) compound and sectioned at 10–12 μm using a cryostat.

Hematoxylin and Eosin Staining: Paraffin sections were dewaxed, rehydrated, and stained with Harris hematoxylin solution for three minutes. Sections were then counterstained with eosin alcoholic solution for one minute, dehydrated through graded ethanol, cleared in xylene, and mounted with neutral balsam.

Von Kossa Staining: Sections were incubated with 5% silver nitrate solution for 30 minutes under ultraviolet light. After washing, sections were treated with 5% sodium thiosulfate solution for two minutes to remove unreacted silver and counterstained with nuclear fast red. Calcific deposits appeared as black precipitates.

Alizarin Red S Staining (Tissue): Frozen sections were air-dried, fixed with 4% paraformaldehyde for five minutes, and stained with 2% Alizarin Red S solution (pH 4.2) for two minutes. Excess dye was removed, and sections were dehydrated in acetone and acetone–xylene, cleared in xylene, and mounted with DPX mounting medium.

Modified Masson's Trichrome Staining: Sections were stained with Weigert's iron hematoxylin for five minutes, followed by Biebrich scarlet–acid fuchsin solution for ten minutes. Sections were differentiated in phosphomolybdic–phosphotungstic acid solution and counterstained with aniline blue. Collagen fibers appeared blue, and cytoplasm appeared red.

Picrosirius Red Staining: Sections were stained with Picrosirius Red solution (Sirius Red F3B in saturated picric acid) for one hour at room temperature, washed in acidified water, dehydrated, cleared, and mounted. Collagen fibers appeared red under bright-field microscopy and exhibited birefringence under polarized light microscopy.

The percentage of the positively stained area relative to the total tissue section area was quantified using ImageJ software by an investigator blinded to the experimental groups.

**Immunofluorescence Staining**

For cell immunofluorescence, cells were washed twice with PBS and fixed with 4% paraformaldehyde for 15 minutes. For tissue immunofluorescence, paraffin sections were dewaxed in xylene and rehydrated through a graded ethanol series to distilled water prior to fixation. All samples were permeabilized with 0.2% Triton X-100 for ten minutes, blocked with 5% BSA (Biofrox, 4240GR100) for 30 minutes, and incubated overnight at 4 °C with primary antibodies at the dilutions listed in Table S3, followed by incubation with secondary antibodies for one hour at room temperature. All samples were washed three times with PBS, counterstained with DAPI for five minutes, and mounted with ProLong Glass Antifade Mountant (Invitrogen, P36982). Images were acquired on a laser scanning confocal microscope (LSM980, Zeiss). Three-dimensional reconstructions of confocal images were generated from z-axis image stacks using Imaris software (version 10.1.1). Primary and secondary antibodies are listed in Table S3.

For quantitative analysis, fluorescence intensity was measured within manually defined regions of interest (ROIs) corresponding to individual cells or valve leaflet sections using ImageJ (NIH, v1.54). p16^INK4A^ and p21 immunofluorescence data from human valve sections were expressed as median nuclear fluorescence intensity; all other immunofluorescence analyses were expressed as mean fluorescence intensity (MFI). A minimum of six randomly selected fields per section and at least six sections per group were analyzed, with background fluorescence subtracted using an unstained control.

**Stimulated Emission Depletion Super-Resolution Microscopy**

hVICs were immunostained as described above. Imaging was performed on a Leica TCS SP8 STED 3X system with a 100× oil immersion objective (NA 1.4).

**Animal Models**

All animal experiments were performed in strict compliance with the Guide for the Care and Use of Laboratory Animals (National Research Council, 2011) and were approved by the Institutional Animal Care and Use Committee of West China Hospital (Approval No. 20240311055). Mice were maintained in a specific pathogen-free facility under a 12-hour light/dark cycle with free access to food and water. All surgical procedures were performed under isoflurane anesthesia (3% induction, 1.5–2% maintenance in room air). Anesthetic depth was monitored throughout by pedal withdrawal reflex. Post-operative analgesia was provided by subcutaneous meloxicam (5 mg/kg). At experimental endpoints, deep anesthesia was confirmed by loss of pedal reflex, followed by cervical dislocation as the method of euthanasia in accordance with the Guidelines for the Euthanasia of Animals: 2020 Edition issued by the American Veterinary Medical Association (AVMA, 2020).

Aortic Wire Injury (AWI) Model: Wild-type C57BL/6J mice (8–10 weeks old, both sexes) and systemic Gas6-knockout (Gas6^⁻/⁻^) mice were subjected to AWI surgery. Gas6^⁻/⁻^ mice were obtained from Cyagen and genotyped by PCR using ear notch DNA (primer sequences in Table S5). Briefly, mice were anesthetized with 2% isoflurane. A 0.014-inch guidewire was inserted into the left ventricle via the right common carotid artery and passed across the aortic valve to induce mechanical injury. Sham-operated mice underwent identical surgical procedures without wire insertion. Disease progression was evaluated at four weeks post-surgery by echocardiography and histological analysis.

Diet-Induced CAVD Model: male ApoE⁻/⁻ mice (8 weeks old; Cyagen) were fed a purified high-fat diet providing 40% of energy from fat and supplemented with 1.25% cholesterol and 0.5% sodium cholate (Xietong Shengwu, XT109C) for 24 weeks to induce aortic valve calcification. Age-matched ApoE⁻/⁻ mice fed standard chow served as controls. Following the dietary intervention, tissues were harvested for immunofluorescence analysis of Gas6 and RUNX2.

**Echocardiography**

Transthoracic echocardiography was performed using a Vevo 3100 Imaging System (Fujifilm VisualSonics) with a 40-MHz linear array ultrasound transducer (MX550D, VisualSonics Inc.). Mice were anesthetized with 1.5–2% isoflurane in room air and placed on a heated platform to maintain body temperature at 37 °C. Aortic valve morphology and leaflet motion were assessed in the parasternal long-axis view. Peak transvalvular jet velocity (Vmax) and mean pressure gradient were measured from the **suprasternal aortic arch view** using pulsed-wave Doppler. Effective orifice area (EOA) was calculated using the continuity equation: EOA = (LVOT CSA × LVOT VTI) / AV VTI, where left ventricular outflow tract (LVOT) cross-sectional area (CSA) was derived from LVOT diameter, and velocity-time integral (VTI) was measured at the LVOT and aortic valve, respectively. All measurements were performed by an investigator blinded to the experimental groups, and the average of three consecutive cardiac cycles was reported.

**Statistical Analysis**

Experimental data are presented as mean ± SEM unless otherwise indicated; baseline characteristics in Table S1 are presented as mean ± SD for continuous variables and n (%) for categorical variables. For in vitro experiments, each data point represents an independent biological replicate derived from a separate patient donor (n = 6 donors per experiment). For in vivo experiments, each data point represents an independent animal (n = 6 per group). Both sexes were included in the aortic wire injury model. Sex-by-treatment interactions were assessed using two-way ANOVA; no significant sex-by-treatment interactions were observed, and data from male and female mice were therefore pooled. The diet-induced ApoE⁻/⁻ model employed male mice exclusively owing to estrogen-mediated atheroprotection in females. Normality was verified by the Shapiro–Wilk test. Statistical comparisons between two groups were performed using two-tailed unpaired Student's t-test. Categorical variables were compared using Fisher's exact test. Comparisons among three or more groups were analyzed by one-way ANOVA followed by Tukey's post hoc test. All statistical analyses were performed using GraphPad Prism (v10.6). A two-sided P value < 0.05 was considered statistically significant.

**Supplementary References**

American Veterinary Medical Association. (2020). *AVMA guidelines for the euthanasia of animals: 2020 edition*. https://www.avma.org/sites/default/files/2020-02/Guidelines-on-Euthanasia-2020.pdf

National Research Council. (2011). *Guide for the care and use of laboratory animals (8th ed.)*. The National Academies Press. https://doi.org/10.17226/12910

Praz, F., Borger, M. A., Lanz, J., Marin-Cuartas, M., Abreu, A., Adamo, M., ... ESC/EACTS Scientific Document Group. (2025). 2025 ESC/EACTS Guidelines for the management of valvular heart disease. *European Heart Journal, 46*, 4635–4736. https://doi.org/10.1093/eurheartj/ehaf194

Shao, R., Li, J., Wu, F., Meng, W., Li, C., Huang, F., ... Chen, M. (2026). Tom70 prevents fibrotic activation of aortic valve interstitial cells via EPA-activated autophagic flux. *Biochimica et Biophysica Acta (BBA) - Molecular Basis of Disease, 1872*, 168063. https://doi.org/10.1016/j.bbadis.2025.168063

**Supplementary Figures**

*Supplementary Figures S1, S2, S3, S5, and S6 correspond to main text Figures 1, 2, 3, 5, and 6, respectively.*

**Supplementary Figure S1. Osteogenic stimulation enhances MDV biogenesis and characteristic mitochondrial morphological remodeling, related to Figure 1.**

(A) Quantitative analysis of the relative protein expression levels of RUNX2, OPN, and BMP2 from the immunoblot data presented in Figure 1H.

(B) Schematic illustration of the in vitro calcification-inducing culture and MDV isolation protocol.

(C and D) Representative nano-flow cytometry plots (C) and quantitative analysis (D) of MDVs secreted by hVICs treated with increasing intensities of pro-osteogenic medium (0.5×, 1.0×, and 2.0× OM), demonstrating a dose-dependent increase in MDV release. Values were normalized to 0.5× OM.

(E) Quantitative analysis of the relative protein expression levels of RUNX2, OPN, and BMP2 from the immunoblot data presented in Figure 1L. Values were normalized to CM.

(F) Stimulated emission depletion (STED) super-resolution microscopy images comparing mitochondrial networks in CM- and OM-cultured hVICs. Scale bars, 6 μm (upper); 1 μm (lower).

(G) Representative transmission electron microscopy (TEM) images of in vitro cultured hVICs showing the ultrastructure of donut-shaped mitochondria induced by OM. Scale bars, 6 μm (left); 0.6 μm (right).

(H) Live-cell imaging of mitochondrial morphology in hVICs cultured under CM or OM conditions. Scale bars, 30 μm (left); 3 μm (right).

(I) Quantitative analysis comparing the percentage of donut-shaped mitochondria between CM and OM groups.

(J and K) Representative nano-flow cytometry plots (J) and quantitative analysis (K) of MDVs released into conditioned medium by hVICs at passages 1, 3, and 7 (P1, P3, and P7, respectively). Values in (K) were expressed as fold changes and normalized to P1, which was set to 1. MDV release progressively increased across hVIC passages P1, P3, and P7.

Data are presented as mean ± SEM (n = 6 independent experiments). P values were determined by two-tailed unpaired Student's t-test for comparisons between two groups, and by one-way ANOVA followed by Tukey's post hoc test for multiple comparisons. For panel K, P = 0.0459 for P1 versus P3 and P < 0.0001 for P3 versus P7.

**Supplementary Figure S2. Senescence-associated marker changes in MDV-exposed recipient hVICs and Gas6 expression in HFD-induced murine valves, related to Figure 2.**

(A and B) Representative immunoblots (A) and densitometric quantification (B) of p16^INK4A^ and p21 protein expression in recipient hVICs exposed to MDV preparations derived from CM- or OM-treated donor hVICs. GAPDH served as the loading control.

(C) Representative senescence-associated β-galactosidase (SA-β-gal) staining images of recipient hVICs exposed to MDV preparations derived from CM- or OM-treated donor hVICs. Lower panels show higher-magnification views. Scale bar, 200 μm (lower panels).

(D and E) Representative immunoblots (D) and densitometric quantification (E) of p16^INK4A^ and p21 protein expression in recipient hVICs exposed to MDV preparations derived from nonCAVD or CAVD human valve tissues. GAPDH served as the loading control.

(F) Representative SA-β-gal staining images of recipient hVICs exposed to MDV preparations derived from nonCAVD or CAVD human valve tissues. Lower panels show higher-magnification views. Scale bar, 200 μm (lower panels).

(G and H) Representative immunofluorescence co-staining images (G) and quantitative analysis of mean fluorescence intensity (H) for Gas6 (red) and RUNX2 (green) in aortic valve leaflets from ApoE⁻/⁻ mice fed normal chow or a high-fat diet (HFD). Nuclei were counterstained with DAPI (blue). Scale bar, 50 μm.

Data are presented as mean ± SEM (n = 6 independent experiments for quantitative hVIC analyses; n = 6 mice per group for murine immunofluorescence analysis). For densitometric and mean fluorescence intensity quantification, values were normalized to the first group. P values were determined by two-tailed unpaired Student's t-test.

**Supplementary Figure S3. Effects of Gas6 manipulation on hVIC calcification, osteogenic programming, and senescence-associated features, related to Figure 3.**

(A) Representative image of non-calcified human aortic valve leaflets used for primary hVIC isolation.

(B–F) Gas6 and osteogenic marker expression in hVICs transduced with control adenovirus (AdCtrl) or Gas6-expressing adenovirus (AdGas6) and cultured in control medium (CM) or pro-osteogenic medium (OM). (B) Representative immunoblots of Gas6, RUNX2, OPN, and BMP2. GAPDH served as the loading control. (C–F) Densitometric quantification of Gas6 (C), RUNX2 (D), OPN (E), and BMP2 (F).

(G) Representative whole-well and higher-magnification images of Alizarin Red S staining in hVICs transduced with AdCtrl or AdGas6 and cultured in CM or OM for 21 days. Scale bar, 200 μm (lower panels).

(H–J) Senescence-associated protein expression following Gas6 knockdown. (H) Representative immunoblots of p21 and p16^INK4A^ in hVICs treated with non-targeting control siRNA (siCtrl) or Gas6-targeting siRNA (siGas6) under CM and OM conditions. GAPDH served as the loading control. (I and J) Densitometric quantification of p21 (I) and p16^INK4A^ (J).

(K) Representative senescence-associated β-galactosidase (SA-β-gal) staining images of siCtrl- and siGas6-treated hVICs under CM and OM conditions. Upper panels show whole-well images, and lower panels show higher-magnification views. Scale bar, 200 μm (lower panels).

(L–N) Senescence-associated protein expression following Gas6 overexpression. (L) Representative immunoblots of p21 and p16^INK4A^ in hVICs transduced with AdCtrl or AdGas6 under CM and OM conditions. GAPDH served as the loading control. (M and N) Densitometric quantification of p21 (M) and p16^INK4A^ (N).

(O) Representative SA-β-gal staining images of AdCtrl- and AdGas6-transduced hVICs under CM and OM conditions. Upper panels show whole-well images, and lower panels show higher-magnification views. Scale bar, 200 μm (lower panels).

Quantified immunoblot data are presented as mean ± SEM (n = 6 independent experiments for panels C–F, I, J, M, and N). Values were normalized to the first group. P values were determined by one-way ANOVA with Tukey's post hoc test.

**Supplementary Figure S5. Densitometric quantification of DRP1 scission machinery components in siCtrl and siGas6 hVICs and osteogenic marker expression in recipient hVICs, related to Figure 5.**

(A–E) Densitometric quantification of the p-DRP1/DRP1 ratio (A), MIRO1 (B), MIRO2 (C), MFF (D), and FIS1 (E) from immunoblot data presented in Figure 5B.

(F–H) Densitometric quantification of RUNX2 (F), OPN (G), and BMP2 (H) in recipient hVICs from immunoblot data presented in Figure 5G.

Data are presented as mean ± SEM (n = 6 independent experiments). For densitometric quantification, protein expression levels were normalized to the first group. P values were determined by one-way ANOVA with Tukey's post hoc test.

**Supplementary Figure S6. Quantitative and gain-of-function validation of the Gas6–Ca²⁺–CaMKII axis, related to Figure 6.**

(A) Quantification of mean Fluo-8 fluorescence intensity in siCtrl and siGas6 hVICs under CM and OM conditions, normalized to siGas6 CM group (related to Figure 6B).

(B and C) Representative Fluo-8 fluorescence images (B) and quantification of mean fluorescence intensity (C) in AdCtrl- and AdGas6-transduced hVICs under OM conditions, normalized to the AdCtrl CM group. Scale bars, 20 μm.

(D) Densitometric quantification of the p-CaMKII/CaMKII ratio following Gas6 knockdown (related to Figure 6C).

(E and F) Representative immunoblots (E) and densitometric quantification of the p-CaMKII/CaMKII ratio (F) in AdGas6-transduced hVICs under OM conditions treated with or without Mdivi-1.

(G–I) Densitometric quantification of RUNX2 (G), OPN (H), and BMP2 (I) in recipient hVICs following the MDV transfer assay (related to Figure 6H).

(J) Densitometric quantification of p-CaMKII/CaMKII ratio, p-DRP1/DRP1 ratio, MIRO1 and MIRO2 following KN-93 treatment in AdGas6-transduced hVICs (related to Figure 6I).

Data are presented as mean ± SEM (n = 6 independent experiments). For densitometric quantification, protein expression levels were normalized to the first group. P values were determined by one-way ANOVA with Tukey's post hoc test.

**Supplementary Videos**

**Supplementary Video 1. Rotational view of a three-dimensional reconstruction from confocal z-stacks, related to Figure 1N.** Rotational view of the static three-dimensional reconstruction of a TOM20⁺/PDH⁺ donut-shaped mitochondrial structure with an attached vesicular bud in an OM-treated hVIC. Rotation of the reconstructed volume is shown for spatial visualization and does not represent time-lapse imaging.

**Supplementary Video 2. Multi-angle rotational view of a three-dimensional TEM reconstruction, related to Figure 1O.** Multi-angle rotational view of the static three-dimensional reconstruction corresponding to the donut-shaped mitochondrial profile in human CAVD valve tissue shown in Figure 1O. Rotation of the reconstructed volume is shown for spatial visualization and does not represent time-lapse imaging.

**Supplementary Video 3. Gas6 depletion does not impair formation of donut-shaped mitochondria, related to Figure 4D.** Time-lapse imaging of mitochondria (PKmito Deep Red) in siGas6 hVICs under OM conditions. Despite Gas6 knockdown, elongated mitochondria undergo spontaneous curling and self-fusion to form closed donut-shaped structures. Images were acquired at 25-second intervals. Scale bar, 1 μm.

**Supplementary Video 4. Time-resolved three-dimensional reconstruction of mitochondrial curling after Gas6 depletion, related to Figure 4E.** The continuous reconstruction was generated from sequential confocal z-stacks acquired at each time point for the same mitochondrial-curling event shown in Figure 4D; Figure 4E shows three selected time points from this reconstruction.

**Supplementary Video 5. Active MDV budding and release in control cells, related to Figure 4H.** Time-lapse imaging of mitochondria (PKmito Deep Red) in siCtrl hVICs under OM conditions, demonstrating active MDV budding from donut-shaped mitochondria with successful membrane detachment and vesicle release. Images were acquired at 25-second intervals. Scale bar, 1 μm.

**Supplementary Video 6. "Stalled budding" phenotype in Gas6-depleted cells, related to Figure 4J.** Time-lapse imaging of mitochondria (PKmito Deep Red) in siGas6 hVICs under OM conditions. Despite the presence of intact donut-shaped precursors, nascent MDVs remain persistently tethered to parent mitochondria by elongated membrane necks without completing membrane scission. Images were acquired at 25-second intervals. Scale bar, 1 μm.

**Supplementary Video 7. Time-resolved three-dimensional comparison of MDV detachment and persistent tethering, related to Figures 4I and K.** The continuous reconstructions were generated from sequential confocal z-stacks acquired at each time point for the same events shown in Figures 4H and J and are presented side by side. In siCtrl hVICs under OM conditions, the nascent MDV completed terminal detachment/release; in siGas6 hVICs under OM conditions, the nascent MDV remained persistently tethered to the parent mitochondrion without completing membrane scission. Figures 4I and K each show three selected time points from the corresponding reconstruction.

**Supplementary Video 8. Active MDV budding and release in Gas6-overexpressing cells, related to Figure 5.** Time-lapse imaging of mitochondria (PKmito Deep Red) in AdGas6-transduced hVICs under OM conditions. Donut-shaped mitochondria exhibit active MDV budding events culminating in successful membrane scission and vesicle release. Images were acquired at 25-second intervals. Scale bar, 1 μm.

**Supplementary Video 9. DRP1 inhibition impairs MDV detachment in Gas6-overexpressing cells, related to Figure 5.** Time-lapse imaging of mitochondria (PKmito Deep Red) in AdGas6-transduced hVICs under OM conditions treated with Mdivi-1. Nascent MDVs remain persistently tethered to parent mitochondria without completing membrane scission, phenocopying the "stalled budding" observed upon Gas6 knockdown. Images were acquired at 25-second intervals. Scale bar, 1 μm.

**Supplementary Tables**

**Table S1.** Clinical Characteristics of Patients Providing Human Aortic Valve Tissue

| **Characteristic** | **nonCAVD (n = 12)** | **CAVD (n = 12)** | **P value** |
| --- | --- | --- | --- |
| **Demographics** |  |  |  |
| Age, years | 64.4 ± 2.6 | 65.4 ± 4.0 | 0.474 |
| Sex, male, n (%) | 6 (50.0) | 6 (50.0) | 1.000 |
| **Aortic Valve Morphology** |  |  |  |
| Tricuspid aortic valve, n (%) | 9 (75.0) | 9 (75.0) | 1.000 |
| Bicuspid aortic valve, n (%) | 3 (25.0) | 3 (25.0) | 1.000 |
| **Echocardiographic Parameters** |  |  |  |
| Peak transvalvular velocity (Vmax), m/s | 1.4 ± 0.3 | 4.5 ± 0.7 | **<0.001** |
| LVEF, % | 63.8 ± 3.2 | 63.8 ± 5.8 | 0.966 |
| **Comorbidities** |  |  |  |
| Hypertension, n (%) | 2 (16.7) | 2 (16.7) | 1.000 |
| Diabetes mellitus, n (%) | 2 (16.7) | 3 (25.0) | 1.000 |
| Chronic kidney disease, n (%) | 0 (0.0) | 0 (0.0) | — |
| Dyslipidemia, n (%) | 2 (16.7) | 2 (16.7) | 1.000 |
| Atrial fibrillation, n (%) | 3 (25.0) | 4 (33.3) | 1.000 |
| **Laboratory Parameters** |  |  |  |
| eGFR, mL/min/1.73 m² | 87.9 ± 8.4 | 90.1 ± 10.0 | 0.566 |
| Serum creatinine, μmol/L | 82.2 ± 14.8 | 83.1 ± 11.1 | 0.866 |
| Ionized calcium (Ca²⁺), mmol/L | 1.17 ± 0.08 | 1.14 ± 0.08 | 0.367 |
| Serum phosphate, mmol/L | 1.10 ± 0.11 | 1.06 ± 0.12 | 0.374 |
| ALP, U/L | 81.1 ± 19.9 | 78.0 ± 21.2 | 0.717 |
| Total cholesterol, mmol/L | 3.7 ± 0.8 | 3.9 ± 0.5 | 0.460 |
| LDL-cholesterol, mmol/L | 2.4 ± 0.6 | 2.3 ± 0.7 | 0.783 |
| HbA1c, % | 5.4 ± 0.8 | 5.5 ± 0.9 | 0.824 |
| **Medications** |  |  |  |
| Statin, n (%) | 2 (16.7) | 2 (16.7) | 1.000 |

Data are presented as mean ± SD for continuous variables and n (%) for categorical variables.

Continuous variables were compared using unpaired Student's t-test; categorical variables were compared using Fisher's exact test. All tests were two-sided; P < 0.05 was considered statistically significant.

— indicates that statistical comparison was not applicable because no events occurred in either group.

Abbreviations: CAVD, calcific aortic valve disease; eGFR, estimated glomerular filtration rate; ALP, alkaline phosphatase; HbA1c, glycated hemoglobin; LDL, low-density lipoprotein; LVEF, left ventricular ejection fraction; SD, standard deviation; Vmax, peak transvalvular velocity.

Severe aortic stenosis was defined as Vmax ≥ 4.0 m/s according to current ESC/EACTS guidelines (Praz et al., 2025).

Of the 12 patients per group, 6 provided valve tissue for ex vivo hemodynamic perfusion and tissue-derived MDV experiments. The remaining 6 patients per group provided tissue for human histological and molecular analyses, including assessments of senescence-associated features. Primary hVICs used in all in vitro experiments were isolated exclusively from the 6 nonCAVD donors in the second patient subset.

**Table S2. Primary antibodies for immunoblotting**

| Antigen | Supplier | Host species | Dilution |
| --- | --- | --- | --- |
| RUNX2 | Cell Signaling, 12556 | Rabbit | 1:1000 |
| OPN | Abclonal, A19092 | Rabbit | 1:1000 |
| BMP2 | Abcam,  ab243043 | Rabbit | 1:1000 |
| CD9 | Proteintech, 20597-1-AP | Rabbit | 1:2000 |
| CD63 | Proteintech, 25682-1-AP | Rabbit | 1:1000 |
| β-actin | Proteintech, 66009-1-Ig | Mouse | 1:50000 |
| GAPDH | Proteintech, 60004-1-Ig | Mouse | 1:50000 |
| Gas6 | Cell Signaling, 67202 | Rabbit | 1:1000 |
| p16^INK4A^ | Proteintech, 10883-1-AP | Rabbit | 1:1000 |
| p21 | Proteintech, 10355-1-AP | Rabbit | 1:1000 |
| Phospho-DRP1 (Ser616) | Cell Signaling, 3455 | Rabbit | 1:1000 |
| DRP1 | BD Pharmingen, 611112 | Mouse | 1:250 |
| MIRO1 | Proteintech, 84055-5-RR | Rabbit | 1:5000 |
| MIRO2 | Proteintech, 11237-1-AP | Rabbit | 1:2000 |
| MFF | Proteintech, 17090-1-AP | Rabbit | 1:5000 |
| FIS1 | Proteintech, 10956-1-AP | Rabbit | 1:2000 |
| Phospho-CaMKII (Thr286) | Cell Signaling, 12716 | Rabbit | 1:1000 |
| CaMKII | Cell Signaling, 4436 | Rabbit | 1:1000 |

**Table S3. Antibodies for immunofluorescence staining**

| Antigen | Supplier | Host species | Dilution |
| --- | --- | --- | --- |
| TOM20 (ABflo® 488-conjugated) | ABclonal, A26783 | Rabbit | 1:100 |
| PDH | Proteintech, 18068-1-AP | Rabbit | 1:100 |
| RUNX2 | Santa Cruz Biotechnology, sc-390351 | Mouse | 1:50 |
| BMP2 | Abcam, ab214821 | Rabbit | 1:100 |
| Gas6 | Cell Signaling, 67202 | Rabbit | 1:100 |
| p16^INK4A^ | Proteintech, 10883-1-AP | Rabbit | 1:1000 |
| p21 | Proteintech, 10355-1-AP | Rabbit | 1:200 |
| Alexa Fluor Plus 647 | Invitrogen, A32733 | Goat | 1:1000 |
| Alexa Fluor Plus 555 | Invitrogen, A32732 | Goat | 1:1000 |

**Table S4. Antibodies for nano-flow cytometry**

| Antigen | Supplier | Host species | Dilution |
| --- | --- | --- | --- |
| TOM20 | Abclonal, A26783 | Rabbit | 1:50 |

**Table S5. Primer sequences for Gas6 mouse genotyping PCR**

| Gene | Forward Sequence (5′–3′) | Reverse Sequence (5′–3′) |
| --- | --- | --- |
| Gas6 | ACCGTCGCTTAGCCAGGATG | CAGCCGATTAGACACAAGTCAAAGC |

**Table S6. siRNA Sequences**

|  | Sense (5′–3′) | Antisense (5′–3′) |
| --- | --- | --- |
| siCtrl | UUCUCCGAACGUGUCACGUTT | ACGUGACACGUUCGGAGAATT |
| siGas6 | CCACGAAGAAACUCAAGAATT | UUCUUGAGUUUCUUCGUGGTT |
